# Postnatal persistence of hippocampal Cajal-Retzius cells has a crucial role in the establishment of the hippocampal circuit

**DOI:** 10.1242/dev.202236

**Published:** 2024-01-09

**Authors:** Ingvild Lynneberg Glærum, Keagan Dunville, Kristian Moan, Maike Krause, Nicola Pietro Montaldo, Hinako Kirikae, Maximiliano Jose Nigro, Pål Sætrom, Barbara van Loon, Giulia Quattrocolo

**Affiliations:** ^1^ Kavli Institute for Systems Neuroscience and Center for Algorithms of the Cortex, Norwegian University of Science and Technology (NTNU), Trondheim 7491, Norway; ^2^ Mohn Research Center for the Brain, Norwegian University of Science and Technology (NTNU), Trondheim 7491, Norway; ^3^ Department of Clinical and Molecular Medicine, Norwegian University of Science and Technology (NTNU), Trondheim 7491, Norway

**Keywords:** Cajal-Retzius cells, Hippocampus, Development, Spines

## Abstract

Cajal-Retzius (CR) cells are a transient neuron type that populate the postnatal hippocampus. To understand how the persistence of CR cells influences the maturation of hippocampal circuits, we combined a specific transgenic mouse line with viral vector injection to selectively ablate CR cells from the postnatal hippocampus. We observed layer-specific changes in the dendritic complexity and spine density of CA1 pyramidal cells. In addition, transcriptomic analysis highlighted significant changes in the expression of synapse-related genes across development. Finally, we were able to identify significant changes in the expression levels of latrophilin 2, a postsynaptic guidance molecule known for its role in the entorhinal-hippocampal connectivity. These findings were supported by changes in the synaptic proteomic content in CA1 stratum lacunosum-moleculare. Our results reveal a crucial role for CR cells in the establishment of the hippocampal network.

## Teaser

**Highlighted Article:** By ablating hippocampal Cajal-Retzius cells in early postnatal development, we demonstrate severe impairments in the development of dendrites and spines of CA1 pyramidal cells, and in synapse-related genes and proteins.

## INTRODUCTION

The hippocampal formation is a brain structure primarily associated with memory formation, learning and spatial representation (Buzsáki and Moser, 2013). The microcircuits responsible for these functions are refined around birth and during the first few weeks of postnatal development (Alberini and Travaglia, 2017; Donato et al., 2017; Dumas, 2005; Travaglia et al., 2016). During this time, hippocampal neurons undergo changes in morphology, physiological properties and connectivity patterns. These changes are guided by unique genetic programs (Yuste et al., 2020).

Cajal-Retzius (CR) cells are reelin-expressing glutamatergic neurons, the function of which has mainly been attributed to the orchestration of cortical lamination in prenatal development (D'Arcangelo et al., 1995; Gil et al., 2014; Ramón y Cajal et al., 2011). Although most neocortical CR cells disappear soon after birth, 20% of hippocampal CR cells persist for several months (Anstötz et al., 2016; Ma et al., 2014; Supèr et al., 1998). In the hippocampus, CR cells are localized in the molecular layer of the dentate gyrus and the stratum lacunosum-moleculare (SLM) of the CA regions (Anstötz et al., 2016; Del Río et al., 1997). CR cells have extensive axon collaterals that can occasionally be long-ranging, terminating as distantly as the entorhinal cortex (Ceranik et al., 1999; Deng et al., 2007). The long-range projections of CR cells have been proposed to serve as a scaffold for entorhinal fiber innervation of the hippocampus, with CR cells serving as transient targets of entorhinal afferents (Anstötz et al., 2016; Deng et al., 2007; Supèr et al., 1998).

Although CR cells seem to have a clear role during prenatal development, little is known about their function in postnatal hippocampal development. Electrophysiological studies have shown that CR cells are actively integrated in the hippocampal microcircuit, receiving GABAergic inputs and generating glutamatergic outputs to both pyramidal neurons and inhibitory interneurons (Anstötz et al., 2018; Quattrocolo and Maccaferri, 2013, 2014), although their impact on the maturation of the hippocampal circuits remains poorly understood.

To determine the influence of hippocampal CR cells on postnatal development, we have established a model by which we can successfully ablate CR cells exclusively from the postnatal hippocampus. By combining spine analysis, mRNA sequencing and shotgun proteomics, we identified significant and layer-specific changes in cell morphology, in dendritic spines and in the expression of synapse-related genes and synaptic proteins. Our findings indicate that the persistence of CR cells in the postnatal hippocampus is crucial for proper maturation of the hippocampal circuit.

## RESULTS

### Targeting and ablation of hippocampal Cajal-Retzius cells

**Fig. 1.**
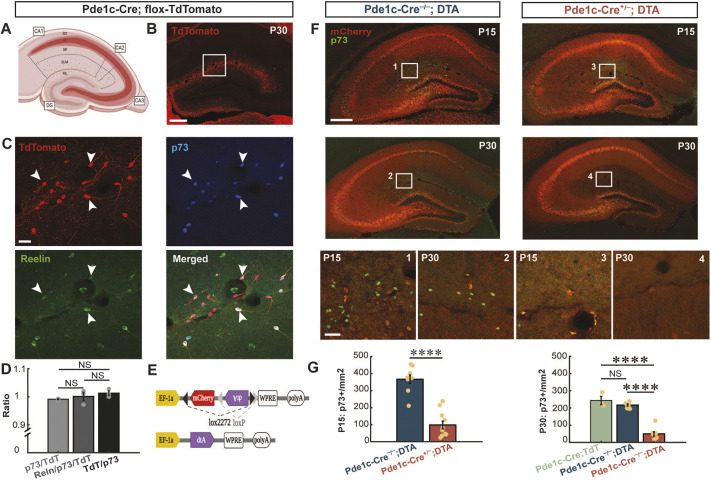
**Targeting and ablation of hippocampal Cajal-Retzius cells.** (A) Schematic depicting an injected hippocampus with layers (SO, SP, SR, SLM and ML), subregions (CA1, CA2, CA3 and DG) and the corresponding location of CR cells (green dots). (B) Image of coronal section depicting tomato^+^ cells in the hippocampus of a Pde1c-Cre^+/−^;flox-TdTomato mice. (C) Magnifications of the area outlined in B depicting immunolabelled TdTomato^+^, p73^+^ and reelin^+^ cells. (D) Bar graph illustrating the ratio of immunolabelled p73^+^, TdTomato^+^ and reelin^+^ cell counts (one-way ANOVA, *n*=3 animals). (E) Schematic of injected virus structure. (F) Examples from P15 (top panels) and P30 (middle panels) of Pde1c-Cre mice injected with AAV-DTA virus. Bottom panels represent high-magnification image of areas outlined above (indicated by numbers 1-4). (G) Comparative analysis of p73^+^ cell densities in Pde1c-Cre^+/−^;flox-TdTomato (P30, *n*=3), Pde1c-Cre^−/−^;DTA (P30, *n*=7; P15, *n*=9) and Pde1c-Cre^+/−^;DTA samples (P30, *n*=11; P15, *n*=8) at P30 (left) (one-way ANOVA) and at P15 (right) (two-sample *t*-tests). Data are group mean±s.e.m. Scale bars: 200 µm in B and F (top and middle); 20 µm in C; 50 µm in F (bottom). *P*≥0.05 (not significant, NS), ****P≤ 0.0001. SO, stratum oriens; SP, stratum pyramidal; SR, stratum radiatum; SLM, stratum lacunosum-molecular; ML, molecular layer.

To determine the role of CR cells in the developing hippocampus, we took advantage of the Pde1c-Cre transgenic mouse line that conditionally expresses Cre in CR cells (Osheroff and Hatten, 2009). First, to confirm the validity of the transgenic line for the hippocampus, we crossed a Pde1c-Cre^+/−^ mouse with an *Ai9* TdTomato reporter mouse and performed immunohistochemical labeling for CR cell-specific markers, reelin and p73 (Meyer et al., 2004). Nearly all TdTomato^+^ cells expressed reelin and p73, and practically all the p73^+^ cells were also TdTomato^+^, with no significant difference between the cell ratio (Fig. 1A-C). Thus, the Pde1c-Cre transgenic mouse line is both selective and specific, labelling all hippocampal CR cells.

To study the contribution of CR cells to the maturation of the postnatal hippocampus, we prematurely induced their death. At P0, we injected a viral vector (pAAV-mCherry-flex-dta) (Wu et al., 2014), encoding mCherry (panneuronaly) and a Cre-dependent diphteria-toxin fragment A (DTA) (Fig. 1E), bilaterally in the dorsal hippocampus of Pde1c-Cre mouse pups. This construct drives the expression of mCherry in all the infected cells, allowing an easy visualization of the injection site (Fig. 1F), whereas only Cre-expressing cells express DTA and undergo cell death. Immunostaining for p73 (an established marker for CR cells) showed a 73% reduction in the number of hippocampal CR cells in Pde1c-Cre^+/−^;DTA at P15 and a 77% reduction at P30 (Fig. 1F,G). CR cell density in control animals was comparable with what we observed in Pde1c-Cre^+/−^;flox-TdTomato mice (uninjected controls), suggesting the injection procedure alone did not directly affect the survival of CR cells (Fig. 1G, right). These results indicate that our targeted approach drastically reduces the postnatal density of CR cells and can thus provide insights on the consequences of their ablation on the development of the hippocampal circuit.

### CR cell ablation alters development of dendritic complexity of CA1 pyramidal cells

We then sought to investigate the impact of CR cell ablation on CA1 pyramidal cells: the principal output neurons of the hippocampus and postsynaptic targets of CR cells (Quattrocolo and Maccaferri, 2014). Although hippocampal pyramidal cells have reached the pyramidal layer at birth (Angevine, 1965), their dendritic arbor is still at a rudimentary stage and continues to develop during the postnatal period (Pokorný and Yamamoto, 1981). Accordingly, the absence of CR cells during this period would likely perturb dendritic development of CA1 pyramidal cells, as has been shown for neocortical pyramidal cells when decreasing CR cell density (de Frutos et al., 2016).

**Fig. 2.**
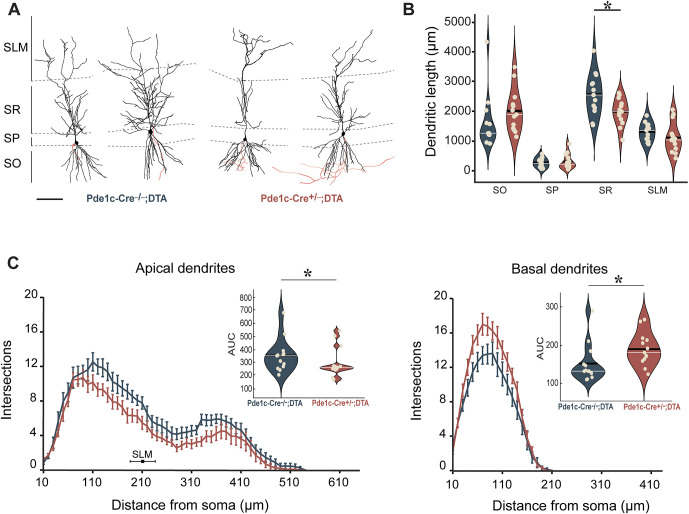
**Morphological analysis of dendritic length and complexity of CA1 pyramidal neurons.** Reconstruction of neurons from Pde1c-Cre^+^/^−^;DTA (*n*=13, from 7 animals) and Pde1c-Cre^−^/^−^;DTA mice (*n*=13, from 10 animals) was based on biocytin filling. (A) Two example cells from each group, with delineation of hippocampal layers and axons in red. Scale bar: 100 μm. (B) Comparison of mean total dendritic length for each hippocampal layer. Pde1c-Cre^−^/^−^;DTA (blue) and Pde1c-Cre^+^/^−^;DTA (red). Raw datapoints are illustrated as circles, scattered for better visualization. The black horizontal lines indicate the mean; the white horizontal lines indicate the median. SR, *P*=0.018 (independent samples *t*-test). Error bar±1 s.e.m. (C) Sholl graphs of dendritic intersections over distance from the soma for the apical and basal dendritic trees. Data are mean±s.e.m. In the apical dendrites graph, the average border of SR and SLM is indicated with a black square±1 s.d. Nested figures depict the area under the curve (AUC) for each group. *P*≥0.05 (not significant), *P<0.05 (Mann-Whitney U-test).

CA1 pyramidal cells were reconstructed with the hippocampal layers SLM, stratum radiatum (SR), stratum pyramidale (SP) and stratum oriens (SO) delineated (Fig. 2A). Differences between the experimental and control groups were observed when comparing dendritic length by layer, i.e. CA1 pyramidal cells from CR cell-ablated mice (Pde1c-Cre^+^/^−^;DTA) were found to have a significantly shorter overall dendritic length in SR (Fig. 2B). Next, we performed a Sholl analysis to further investigate possible differences in dendritic complexity. This revealed deviations in dendritic complexity in both the apical and basal tree of CA1 pyramidal cells in Pde1c-Cre^+^/^−^;DTA mice when compared with controls (Fig. 2C). As the curve of the CA1 pyramidal cells from the Pde1c-Cre^+^/^−^;DTA mice was consistently below those of the experimental group, we can expect that changes in dendritic complexity are present at several distances from the soma, in the apical tree, at both the level of the SR and SLM. Interestingly, in the basal tree, Pde1c-Cre^+^/^−^;DTA mice showed a significantly increased dendritic complexity.

Ablation of hippocampal CR cells resulted in decreased apical dendritic complexity for CA1 pyramidal cells while basal dendritic complexity increased. These findings are in line with what has been reported in studies examining CR cells in neocortical areas (de Frutos et al., 2016) and, interestingly, a similar effect is seen in cortical pyramidal cells when preventing cell death in cortical CR cells (Riva et al., 2019). Thus, our findings suggest an active role for CR cells in the control of CA1 pyramidal cell dendritic complexity.

### Ablation of CR cells disrupts the formation of dendritic spines on CA1 pyramidal cells

Prenatal reduction of CR cells in the barrel cortex also reduced spine density in apical dendrites of cortical pyramidal cells, whereas preventing postnatal CR cell death caused an increased spine density on both apical and basal dendrites (de Frutos et al., 2016; Riva et al., 2019). Therefore, we performed a morphological assessment of dendritic spines at juvenile stages in CR cell-ablated mice.

**Fig. 3.**
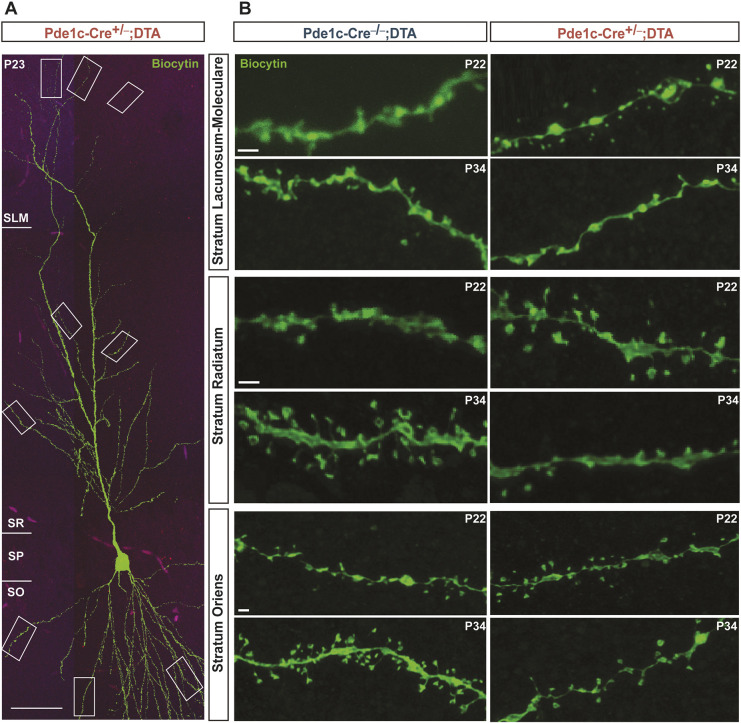
**Layer-specific changes in dendritic spine density of CA1 pyramidal cells.** (A) Example of a biocytin-filled pyramidal cell demonstrating dendrite selection. Each selected dendrite was terminal in the layer of interest (e.g. taken after the last bifurcation). Delineations showing SLM (top), SR (middle) and SO (bottom). (B) Images of terminal dendritic segments in SLM (top), SR (middle) and SO (bottom) of CA1 pyramidal cells in control (*n*=13, from 8 animals) and Pde1c-Cre^+/−^;DTA mice (*n*=13, from 6 animals). Scale bars: 50 µm in A; 1 µm in B.

**Fig. 4.**
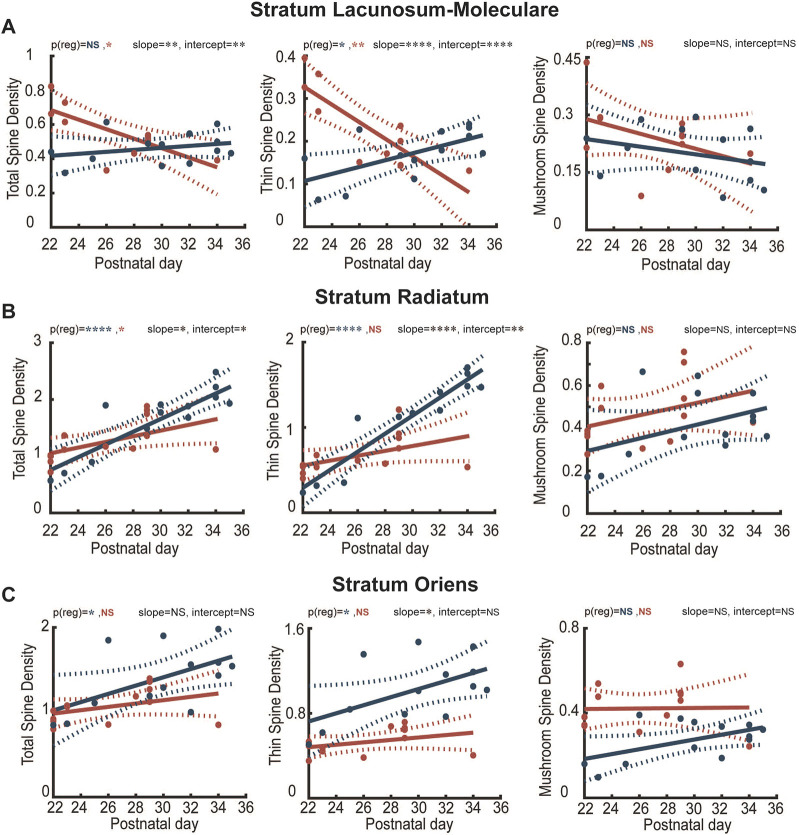
**Correlation between postnatal age and spine density in control and CR cell-ablated mice.** (A) Simple regression models, *p(reg)*, of total (left: Pde1c-Cre^+/−^;DTA: R^2^=0.54 versus Pde1c-Cre^−/−^;DTA: R^2^=0.08), thin (middle: Pde1c-Cre^+/−^;R^2^=0.7 versus Pde1c-Cre^−/−^;DTA: R^2^=0.38) and mushroom (right: Pde1c-Cre^+/−^;DTA: R^2^=0.17 versus Pde1c-Cre^−/−^;DTA: R^2^= 0.09) spine densities in the SLM across developmental time points in control (blue) and Pde1c-Cre^+/−^;DTA (red) mice. (B) Simple regression models of total (left: Pde1c-Cre^+/−^;DTA R^2^=0.77 versus Pde1c-Cre^−/−^;DTA R^2^=.32), thin (middle: Pde1c-Cre^+/−^;DTA R^2^=0.25 versus Pde1c-Cre^−/−^;DTA R^2^=0.89) and mushroom (right: Pde1c-Cre^+/−^;DTA R^2^=0.13 versus Pde1c-Cre^−/−^;DTA R^2^=0.18) spine densities in the SR across developmental time points in control (blue) and Pde1c-Cre^+/−^;DTA (red) mice. (C) Simple regression models of total (left: Pde1c-Cre^+/−^;DTA R^2^=0.15 versus Pde1c-Cre^−/−^;DTA R^2^=0.32), thin (middle: Pde1c-Cre^+/−^;DTA R^2^=0.14 versus Pde1c-Cre^−/−^;DTA R^2^=0.31) and mushroom (right: Pde1c-Cre^+/−^;DTA R^2^ >0.01 versus Pde1c-Cre^−/−^;DTA R^2^=0.28) spine densities in the SO across developmental time points. Post-hoc ANCOVA assessing the difference between all models, indicated as slope and intercept. *P*≥0.05 (not significant), *P<0.05, **P≤0.001, ****P≤0.0001.

Spine formation happens over several weeks, with most immature spines starting to acquire more mature properties from the first postnatal week and onwards (Basarsky et al., 1994; Holtmaat et al., 2005). We quantified the spines present in terminal dendritic segments in the SLM, SR and SO (Fig. 3B), and classified them as filopodia, stubby, thin and mushroom. As the number of filopodia and stubby spines was too low for a proper analysis (data not shown), we concentrated on thin and mushroom spines only. Data were collected from CA1 pyramidal cells filled with biocytin, from animals between P22 and P35. To assess whether the development influenced the quantity of spines, we applied a simple linear regression model to correlate postnatal age with spine density. Dendritic spines of the SLM demonstrated opposite trends between control and experimental group, as total spine density showed an increase at earlier time points, followed by a decrease later in Pde1c-Cre^+/−^;DTA mice, with thin spines demonstrating the most drastic change (Fig. 4A). In SR and SO, spine density is significantly different between the two groups, with the Pde1c-Cre^−/−^;DTA group showing a clear increase that we could not observe in the Pde1c-Cre^+/−^;DTA mice (Fig. 4B,C). As in the SLM, this effect is likely due to significant changes in thin spine densities. No age-related differences were observed in mushroom spines in any of the layers (Fig. 4A-C).

Previous reports showed that as CA1 pyramidal neurons mature, the density of dendritic spines doubles, and that the number of the different spine types change in a non-uniform manner (Harris et al., 1992; Holtmaat et al., 2005). Indeed, our control mice show an increase in spine density as postnatal development proceeds, with different magnitudes across layers, that are comparable with other studies of age-matched controls (Anderson et al., 2017). However, in the absence of CR cells, spine development is severely impaired. In apical dendrites, these impairments can be ascribed to the loss of thin spines over time. These observed layer-specific differences suggest that distinct inputs to CA1 pyramidal cells are differentially affected by CR cell ablation.

### CR cell-dependent changes in gene regulatory networks

**Fig. 5.**
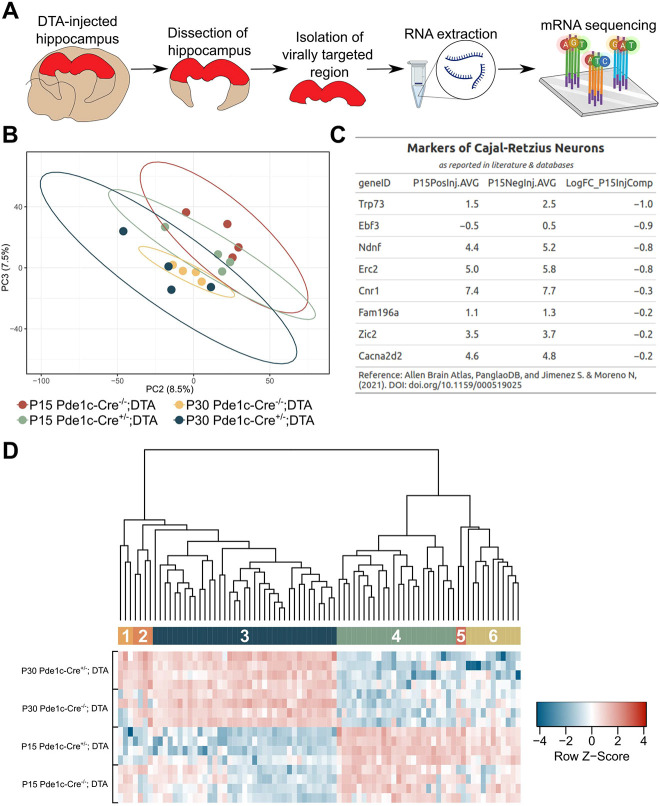
**Bulk mRNA sequencing from dorsal hippocampi of injected mice.** (A) Schematic of mRNA sequencing of injected hippocampi from P15 Pde1c-Cre^−/−^;DTA (*n*=4), P15 Pde1c-Cre^+/−^;DTA (*n*=4), P30 Pde1c-Cre^−/−^;DTA (*n*=4) and P30 Pde1c-Cre^+/−^;DTA (*n*=4). (B) PCA of each group plotted as the second and third principal components as related to the manipulation to ablate CR cells. (C) Table of commonly acknowledged CR cell markers extracted from the literature, including *Ndnf*, *Trp73*, *Ebf3*, *Erc2*, *Zic2*, *Cnr1*, *Erbb4*, *Fam196a* (*Insyn2a*) and *Cacna2d2* (Jiménez and Moreno, 2021). Log_2_FC (LFC) differences were generated by the following equation: [*Gene*]_P15 Pde1c−Cre+/−;DTA_ – [*Gene*]_P15 Pde1c−Cre−/−;DTA_. (D) Heatmap clustering analysis of individual gene expression of individual samples between groups. Genes shown were thresholded at LogFC≥2 and adj *P*-values≤0.05. Genetic and sample clustering were performed using Kendall's correlation method. Colored modules between branches and heatmap cells indicate clustering groups (k=6) driving differences between groups. From left to right, the first (1), second (2) and fifth (5) modules indicate genetic expression differences with respect to CR cell deletion.

To understand molecular underpinnings of the spine changes observed in the CR cell-ablated hippocampus, dorsal hippocampi were microdissected from Pde1c-Cre^−/−^;DTA and Pde1c-Cre^+/−^;DTA groups at P15 and P30. Including two developmental time points allowed us to evaluate the developmental trajectory of gene expression at early (P15) and late (P30) phases of synaptogenesis (Harris et al., 1992). Total RNA was extracted from dissected hippocampal tissue and mRNA was sequenced (Fig. 5A). Fastq reads were mapped to the Mmv79 genome map and its annotated transcriptome using Kallisto; resultant mapped reads per gene were normalized using the TMM method in R. Individual samples were then clustered via PCA analysis and grouped by study design (Fig. 5B). PC1 and PC2 were driven by age-related differences between the P15 and P30 groups, whereas PC2 and PC3 were more related to the manipulation to ablate CR cells. Each group clustered with respect to each other, revealing differences between P15 groups, although P30 Pde1c-Cre^+/−^;DTA was more variable in its clustering and thus did not show separation from the Pde1c-Cre^−/−^;DTA group. Next, we investigated differential gene expression between groups using a contrast matrix. A linear model was then established using the voom variance trend and the normalized gene expression to analyze contrast matrices of all possible comparisons between genotypic, age and treatment groups. A decrease in CR cell-specific markers confirmed CR cell deletion (Fig. 5C). To understand how expression of individual genes contributes to the uniqueness of the transcriptional profile of each study design group, a heatmap analysis was conducted (Fig. 5D). Resultant modules included three gene clusters with noticeable differences between control and CR cell-ablated group (clusters 1, 2 and 5) and one gene cluster of subtle changes (cluster 6). Cluster 1 included *Sh3tc2*, *Fam166b* (*Cimip2b*) and *Hmga1*, cluster 2 included *Gm14440* (*Zfp1009*) *Kansl2*, *Tcte1* and *Tgtp2*, and cluster 5 included *Recql4* and *Socs1*. Genes such as *Socs1* (Baker et al., 2009) and *Tgtp2* (Meijer et al., 2022), encode proteins known to be involved in CNS immune response and are activated by interferon pathways. Genes involved in neuronal development, including neuronal migration (e.g. *Tcte1*; Zur Lage et al., 2019), chromatin remodeling complex genes required for neurogenesis [e.g. *Recql4* (Kohzaki et al., 2021), *Kansl2* (Ferreyra Solari et al., 2016) and *Hmga1* (Vignali and Marracci, 2020)] or myelination genes (e.g. *Sh3tc2*; Arnaud et al., 2009) were significantly changed between control and CR cell-ablated groups at both timepoints (Fig. 5D). Additionally, a neuronal outgrowth-related gene, *Fam166b* (Varga et al., 1995), was downregulated in the P15 Pde1c-Cre^+/−^;DTA group.

**Fig. 6.**
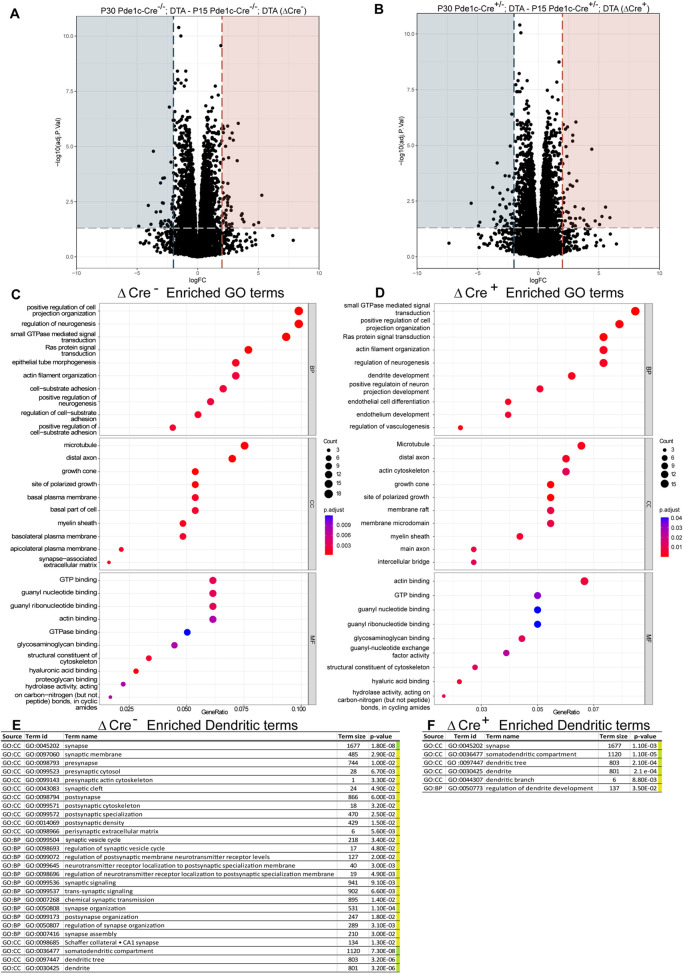
**Volcano plots highlighting gene expression changes.** (A) Volcano plot of the ΔCre^−^ group, demonstrating significant transcriptional changes in ∼70 genes. (B) Volcano plot of the ΔCre^+^ group, demonstrating significant transcriptional changes in 94 genes. Colored shading indicates threshold values at LogFC ≥2 and adj *P*-values≤0.05. Values in blue are significantly downregulated over time, whereas values in red are significantly upregulated. (C) Dot plots of the top 10 GO terms for ‘biological process’ (BP), ‘cellular compartment’ (CC) and ‘molecular function’ (MF) in the ΔCre^−^ group. (D) Dot plots of the top 10 GO terms for ‘biological process’ (BP), ‘cellular compartment’ (CC) and ‘molecular function’ (MF) in the ΔCre^+^ group, highlighting differences in some developmental gene networks. Plots were generated using a *P*-value-ranked gene list of ∼14,555 genes in each group. (E) List of GO terms significantly enriched in ΔCre^−^ group related to dendrites, synapses and/or spines. Twenty-seven unique term enrichments were found to be significantly enriched. (F) List of GO terms significantly enriched in ΔCre^+^ group related to dendrites, synapses and/or spines. Six terms were identified as significantly enriched, five as overlapping with those found in E and one unique to the group: ‘regulation of dendrite development’.

Differentially expressed genes were analyzed by establishing volcano plots of ΔCre^−^ (P30 Pde1c-Cre^−/−^;DTA – P15 Pde1c-Cre^−/−^;DTA) (Fig. 6A) and ΔCre^+^ (P30 Pde1c-Cre^+/−^;DTA – P15 Pde1c-Cre^+/−^;DTA) (Fig. 6B) groups. Significant changes in gene expression were observed in the ΔCre^−^ (Fig. 6C) and ΔCre^+^ (Fig. 6D) groups, further demonstrating that molecular changes mediated by CR cells may occur subtly over this developmental period.

Based on these expression changes, a ranked gene list gene ontology (GO) analysis was performed by sorting genes by descending adjusted *P*-value to account for expression changes masked by somatic mRNA. The top 10 enriched terms in ‘GO biological process’ (BP), ‘GO cellular compartment’ (CC) and ‘GO molecular function’ (MF) were plotted using enrichplot to visualize important gene networks in either the ΔCre^−^ group (Fig. 6C) or the ΔCre^+^ group (Fig. 6D). This analysis revealed conserved ontologies between the developmental groups, such as ‘positive regulation of cell projection organization’, ‘actin filament organization’, ‘distal axon’, ‘growth cone’ and ‘myelin sheath’. However, in addition to decreased gene number and decreased significance in enrichment in most overlapping terms in the ΔCre^+^ group, several ontological terms were not present after CR cell ablation, including ‘positive regulation of neurogenesis’, ‘positive regulation of cell-substrate adhesion’ and ‘synapse-associated extracellular matrix’. Considering the observed changes in spines and dendrites, and the lack of synaptic gene ontological enrichment in the top 10 terms analysis, the same gene lists were then probed for all GO terms related to synapses, spines and dendrites to determine collective processes and cellular compartments in either the ΔCre^−^ (Fig. 6E) or the ΔCre^+^ group (Fig. 6F). Enrichment in the ΔCre^−^ group (Fig. 6E) included 27 terms significantly across several facets of synaptic and dendritic organization, indicating a diversity of active gene networks related to neuronal connectivity. Conversely, the ΔCre^+^ group enrichment across synaptic, spine and dendritic GO terms (Fig. 6F) did not include 22 of the 27 terms found in the ΔCre^−^ group (Fig. 6E) and suggests that some cellular compartments are inherent to the remaining cells but that most of those gene networks are dependent on CR cells, including pre-, post- and trans-synaptic formation and specialization. Interestingly, ‘regulation of dendrite development’ is uniquely enriched in the ΔCre^+^ group, although given the dendritic changes observed in Fig. 2, this is likely to be a negative regulation or the remaining cells are delayed in the timing of their dendritic development. Overall, these results show that the absence of CR cells alters the developmental trajectory of the postnatal hippocampus through gene regulatory networks related to neuronal connectivity and bolsters those spine- and dendrite-related changes observed in Figs 2–4.

**Fig. 7.**
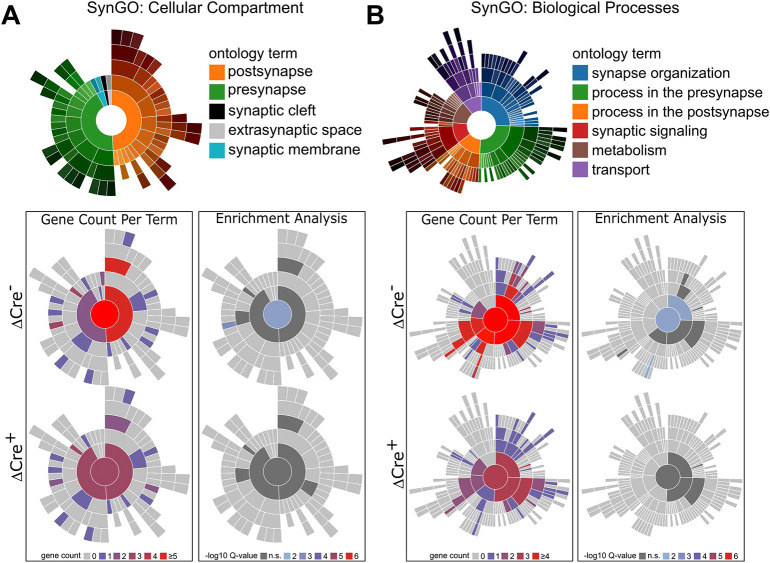
**Gene ontology analysis on synapse-related genes.** (A,B) SynGO analysis of top 200 genes sorted by adjusted *P*-values in the ΔCre groups demonstrating loss of overall gene terms in the cellular compartment (A) and biological processes (B), as well as their significance in enrichment analysis. Spatial legend indicates corresponding location on plots; gene count indicates total term numbers enriched in each cellular compartment; -log_10_Q-values indicate Benjamini-Hochberg corrected *P*-values.

To next investigate the resultant synaptic changes in neurodevelopmental genes and gene networks at the individual synaptic gene level, a second analysis was performed focused on synapse-related ontologies. Ranked gene lists were established for ΔCre^−^ and ΔCre^+^ groups, and were analyzed using SynGO (Koopmans et al., 2019). SynGO analysis returned a significant enrichment in the term ‘synaptic cellular compartment ontology overall’ in the ΔCre^−^ group but a loss in the number of associated terms as well as a loss of significance of enriched terms in the ΔCre^+^ group (Fig. 7A). Likewise, loss and insignificant enrichment of ‘synaptic’ terms in the ΔCre^+^ group was conserved within the ‘biological processes’ term analysis (Fig. 7B). Analysis of individual terms from cellular compartment gene ontology revealed a loss in the number and significance of presynaptic and postsynaptic terms but the most notable loss of enrichment occurred in ‘cytosolic- and membrane-related’ terms at the presynaptic level (Fig. 7A). Similarly, biological process gene ontology analysis revealed a loss in the number and significance of ‘synaptic process’ terms, including ‘synaptic organization’ and ‘modulation of chemical synaptic transmission’ (Fig. 7B). Additionally, a complete loss of ‘synapse assembly’, ‘process in the post-synapse’ and ‘neurotransmitter localization to postsynaptic membrane’ terms was observed in the ΔCre^+^ group. These findings reflect the loss of spines observed in the morphological analysis and further suggest that CR cells mediate connectivity in the postnatal developing hippocampus. The individual genes lost in the ΔCre^+^ group include: *Cpeb1*, a cytoplasmic polyadenylation protein; *Glra2*, glycine receptor subunit alpha 2; *Gpc4*, glypican-4 (cell surface proteoglycan); *Lzts3*, leucine zipper tumor suppressor family member 3; *Map2k1*, mitogen-activated protein kinase kinase 1; *Ncan*, neurocan (axonal pathfinding); *Nptx1*, neuronal pentraxin 1; *Pabpc1*, Polyadenylate-binding protein 1; and *Tnc*, tenascin C. Moreover, when ranked by log fold change and replotted, SynGO analysis demonstrated that *Lphn2* (*Adgrl2*), a unique synaptic marker that facilitates hippocampal connectivity to the entorhinal cortex, is a primary driver of enriched terms. Overall, these findings reinforce the role of CR cells in facilitating synaptic establishment in the developing hippocampus and suggest that their altered expression may further involve alterations within specific microcircuitry functions.

### SLM specific effect of CR-cells ablation

**Fig. 8.**
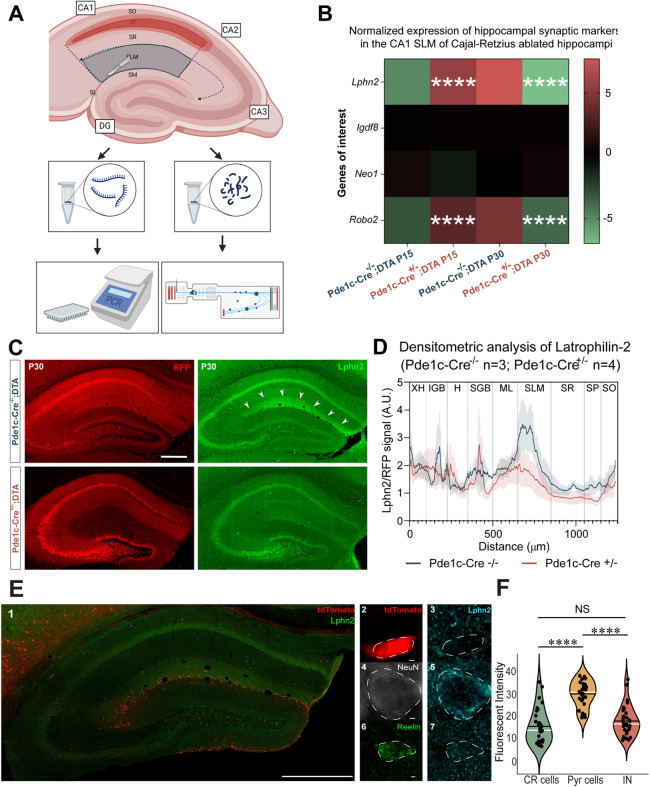
**The SLM-specific effect of CR cell ablation.** (A) Schematic of microdissection of CA1 SLM from hippocampi of P15 Pde1c-Cre^−/−^;DTA (*n*=5), P15 Pde1c-Cre^+/−^;DTA (*n*=6), P30 Pde1c-Cre^−/−^;DTA (*n*=2) and P30 Pde1c-Cre^+/−^;DTA (*n*=5). (B) Heatmap depicting log_2_FC of qRT-PCR-acquired expression of *Adgrl2* (*Lphn2*), *Igsf8*, *Neo1* and *Robo2*. (C) Images of coronal sections depicting Lphn2 expression in Pde1c-Cre^−/−^;DTA and Pde1c-Cre^+/−^;DTA groups at P30. (D) Densitometric analysis of Lphn2 expression in Pde1c-Cre^−/−^;DTA (*n*=3) and Pde1c-Cre^+/−^;DTA (*n*=4) groups at P30. Densitometric signal curves are displayed as mean normalized signal (solid line) and standard error of the mean (shaded curves) were significantly non-zero according to a Deming test (*P*<0.0001). The areas under the ROC curves were significantly different (*P*<0.0001) and individual points were collectively significantly different (*P*<0.0001) across both curves according to a Wilcoxon matched-pairs test. Black vertical lines indicate approximate regions of CA1 and include extrahippocampal (XH), inferior granular blade (IGB), hilar (H), superior granular blade (SGB), molecular layer (ML), stratum lacunosum moleculare (SLM), stratum radiatum (SR), stratum pyramidale (SP) and stratum oriens (SO). (E) Confocal images of hippocampi from Pde1c-Cre^+/−^;flox-TdTomato mice depicting colocalization of TdTomato, Lphn2 (1), reelin and NeuN in CR cells (2 and 3), pyramidal cells (4 and 5) and interneurons (6 and 7). (F) Quantification of fluorescence using an independent samples *t*-test. The black horizontal lines indicate the mean; the white horizontal lines indicate the median. Scale bars: 300 µm in C; 500 µm in E1; 1 µm in E2-E7. *P*≥0.05 (not significant), ****P<0.0001.

Bulk RNAseq analysis of the whole hippocampus revealed significant downregulation of synaptic mRNA after postnatal ablation of CR cells. A deeper analysis of the significantly downregulated genes in the ΔCre^+^ group showed that a unique synaptic marker, *Lphn2*, downregulated in the ΔCre^−^ group, was approximately eight times more downregulated in the ΔCre^+^ group. *Lphn2* encodes the postsynaptic guidance molecule latrophilin 2. In dendritic layers of the hippocampus, latrophilin 2 is exclusively expressed in the CA1 SLM and acts as an axonal guidance molecule for inputs from the entorhinal cortex (Anderson et al., 2017; Donohue et al., 2021; Pederick et al., 2021). To determine whether *Lphn2* is topographically downregulated, CA1 SLM was microdissected from Pde1c-Cre^−/−^;DTA and Pde1c-Cre^+/−^;DTA groups at P15 and P30. Total RNA and protein were extracted from the tissue and qRT-PCR analysis was performed targeting genes specific for CA1 synapses [*Lphn2* (Donohue et al., 2021) and *Robo2* (Blockus et al., 2021)] or CA3 synapses (*Igsf8* and *Neo1*; Apóstolo et al., 2020) to assess specificity of changes (Fig. 8A). qRT-PCR analysis revealed that *Lphn2* and *Robo2* were both significantly upregulated at P15 Pde1c-Cre^+/−^;DTA compared with Pde1c- Cre^−/−^;DTA and, conversely, were significantly downregulated at P30 Pde1c-Cre**^+^**^/−^;DTA compared with Pde1c-Cre^−/−^;DTA (Fig. 8B). No changes were observed in *Igsf8* and *Neo1* expression. The deletion of CR cells inverts the expression trend of synaptic markers responsible for unique inputs to CA1 and may explain the trends observed in spine density (Figs 3 and 4).

Immunohistochemistry confirmed a reduction in *Lphn2* in the SLM at P30 in CR cell-ablated mice (Fig. 8C). Densitometric analysis of the CA1 further confirmed that Lphn2 expression was not only downregulated at P30 in the Pde1c-Cre^+/−^;DTA group compared with the Pde1c-Cre^−/−^;DTA but also that the distribution of Lphn2 was altered in CA1 after early ablation of CR cells. The most notable effect is indeed that Lphn2 is drastically reduced in the SLM, as seen by the peak of Lphn2 expression in the Pde1c-Cre^−/−^ group at ∼750 µm. Moreover, in a descriptive statistical analysis of the curves, only one peak was detected in the Pde1c-Cre^−/−^ group, whereas three diminished peaks were detected in the Pde1c-Cre^+/−^;DTA. The cumulative area under the peaks for Pde1c-Cre^−/−^ and Pde1c-Cre^+/−^ were 813.2 AU and 414.2 AU, respectively, indicating that the distribution is altered and overall signal from Lphn2 is diminished after CR cell ablation at P0. To ensure this reduction could not be caused by the disappearance of CR cells per se, we tested whether CR cells express Lphn2 and could contribute to the reduction in expression levels observed at P30. We quantified the expression of Lphn2 in the somata of CR cells in reelin^+^ interneurons in SLM and CA1 pyramidal cells. To distinguish CR cells, we used brain sections from Pde1c-Cre;flox-TdTomato reporter mice, as in Fig. 1A-D, and no co-expression of TdTomato and Lphn2 was observed. We compared the level of Lphn2 expressed in CR cells with that in CA1 pyramidal cells (Fig. 8E,F), which are known to be Lphn2^+^ (Anderson et al., 2017; Donohue et al., 2021; Pederick et al., 2021) and observed significantly higher levels in the latter group. These data confirmed that the reduction in the Lphn2 levels at P30 cannot be caused by the loss of CR cells per se. In summary, Lphn2 is downregulated both at protein and mRNA level. This demonstrates further that CR cells have a lasting role in synapse maturation.

**Fig. 9.**
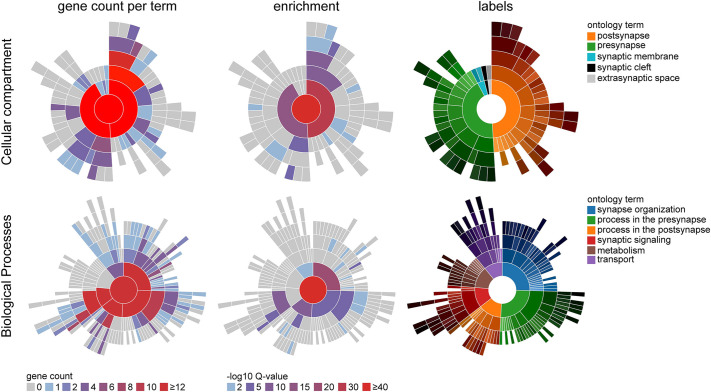
**SynGO analyses of proteins identified as missing in one condition for the ΔCre+ group.** Results as gene count per term (first column) and as enrichment analyses against the ‘brain expressed’ background set of SynGO (second column) are depicted for the cellular compartment (localization, top row) and biological processes (function, bottom row). The third column shows the sunburst structure to indicate the first level of gene ontology term assigned to the respective part of the plot. Gene count indicates the total number of terms identified per gene ontology term and –log10Q-value represents the Benjamini-Hochberg adjusted *P*-values of the enrichment analyses.

Finally, we analyzed the proteomic content of CA1 SLM tissue samples (Fig. 8A) to understand whether other synaptic proteins show changes in their expression within the ΔCre^+^ group. On average, we identified 1600 unique protein groups per sample, of which several hundred could be detected only in the Pde1c-Cre^+/−^;DTA group at P15 or P30. For a detailed analysis of those protein groups, we generated protein lists of the MSstats evaluation, including all identified protein groups that were labeled with ‘one condition missing’ for the ΔCre^+^ group and ΔCre^−^ group. To exclude proteins that would be lost based on the normal developmental trajectory, we excluded all proteins of the ΔCre^−^ group from the ΔCre^+^ group. The resulting protein list was submitted to SynGO analyses to identify synaptic proteins and their localization and function (Fig. 9). In total, 390 proteins were identified to be missing exclusively in the ΔCre^+^ group. The SynGO analyses showed that 70 of those proteins are associated with either synaptic localization and/or synaptic function, and they are exclusively missing from the ΔCre^+^ group at P30. Proteins associated with gene ontology terms for the postsynapse including ‘postsynaptic specialization’ and ‘postsynaptic density’, as well as the presynapse, including ‘presynaptic active zone’ and ‘synaptic vesicles’ were identified for the cellular compartment analyses by SynGO. For the biological function gene ontology, we found terms including processes in the presynapse, such as the ‘synaptic vesicle cycle’, processes in the postsynapse, such as ‘regulation of postsynaptic membrane neurotransmitter receptor levels’, and synaptic signaling processes, including ‘trans-synaptic signaling’, ‘chemical synaptic transmission’ and ‘modulation of chemical synaptic transmission’. The enrichment analyses of those 70 proteins against the brain-expressed background set of SynGO emphasizes that, in particular, proteins associated with the ‘postsynaptic specialization’ and ‘presynaptic active zone’, as well as with ‘synapse organization’, the ‘synaptic vesicle cycle’ and ‘trans-synaptic signaling’, are included in the group of proteins missing from the ΔCre^+^ group. Cross referencing our proteomic SynGO results with available expression data at the Allen mouse brain atlas showed that the missing identified proteins are not solely expressed by CR cells but are rather broadly found in different brain regions. Examples of these proteins are members of the synapsin family, e.g. synapsin 3 (SYN3), that regulate neurotransmitter release (Kao et al., 1998) and synaptotagmin 12 (SYT12), a synaptic vesicle isoform responsible for spontaneous neurotransmitter release (Maximov et al., 2007). Furthermore, we identified exocyst complex component 2 (EXOC2), which is an essential component of the membrane transport machinery and is involved in the docking of exocytic vesicles (Van Bergen et al., 2020). Examples of proteins involved in cell adhesion and synaptogenesis are nectin 1 (NECT1) (Mizoguchi et al., 2002) and the scaffold protein HOMER3 (Hayashi et al., 2009). Interestingly we also identified two subunits of the NMDA receptor (GRIN2A and GRIN2B) in our dataset of proteins missing at P30 in the ΔCre^+^ group, suggesting there are alterations in glutamatergic transmission in CR cell-ablated mice. In summary, our findings reveal that CR cells appear to be crucial for circuitry establishment in the mouse hippocampus during postnatal development.

## DISCUSSION

CR cells are widely understood to have a crucial role in cortical lamination, through the release of reelin (D'Arcangelo et al., 1995; Ma et al., 2014). Their disappearance at early stages of postnatal development in the neocortex (Ma et al., 2014) had often led to their postnatal role in the hippocampus being neglected. However, recent studies have provided more information on hippocampal CR cells. Upon conditional knockout of the vesicular glutamate transporter type 2 (vGlut2) from cortical and hippocampal CR cells Anstötz et al. (2022) reported a reduced spine density and altered synaptic transmission restricted to SLM and the molecular layer of the dentate gyrus. In another recent study, an increase of 30% in CR cells survival was induced in the hippocampus by preventing Bax-dependent apoptosis (Riva et al., 2023). The authors found that an elevated number of CR cells caused increased dendritic complexity and impairments in learning and memory, as well as an increased seizure susceptibility. These findings highlight the importance of the density and connectivity of hippocampal CR cells; however, the manipulations used by the authors were not restricted to the hippocampus or to postnatal development. These approaches not only prevent a clear distinction between the influence of CR cells in pre- versus postnatal development but also cannot exclude the possibility that some of the observed effects are due to well-established alterations in CR cell numbers in other cortical regions (de Frutos et al., 2016; Riva et al., 2019), such as the entorhinal cortex, which is the main input to the hippocampus. To overcome these issues, we decided to combine the use of a specific transgenic mouse line with viral infections, to ablate CR cells exclusively in the postnatal hippocampus. By doing so, we can investigate how development differs between hippocampus and neocortex. Indeed, by inducing early cell death in hippocampal CR cells, we are mimicking the physiological cell death of neocortical CR cells.

In this study, we show that postnatal ablation of hippocampal CR cells leads to: (1) layer-specific alterations in the dendritic morphology and the number and type of dendritic spines on CA1 pyramidal cells; (2) changes in the physiological developmental trajectory of the postnatal hippocampus; (3) downregulation in the expression of synapse-related genes; (4) significant reduction in the mRNA and protein levels of Lhpn2, a gene important for the establishment and maintenance of the entorhinal-hippocampal circuit; and (5) changes in the proteomic content of CA1 SLM in 70 proteins after CR cell ablation.

By inducing early postnatal CR cell death, we were able to reveal a crucial role for CR cells in the postnatal hippocampus. The mechanisms that lead to the observed changes in the hippocampal circuits, however, are yet to be determined. Reelin, the main protein correlated to CR cell function, has been shown to influence synaptogenesis of hippocampal neurons (Bosch et al., 2016; Niu et al., 2008). However, the majority of reelin expression in the postnatal hippocampus has been attributed to GABAergic interneurons (Ramos-Moreno et al., 2006), so it is unlikely that the changes we observe are exclusively due to reduced reelin expression. Nevertheless, we cannot exclude the possibility that the reelin released by CR cells could have specific effects that are not compensated for by the reelin released by interneurons, and therefore contribute to the observed phenotype. CR cells also release glutamate onto both GABAergic cells and CA1 pyramidal neurons (Quattrocolo and Maccaferri, 2014), and, according to the synaptotrophic hypothesis, glutamatergic synaptic transmission regulates synaptogenesis (Cline and Haas, 2008). Our synaptic proteomic analysis showed changes in the expression of proteins involved in synaptic vesicle release and in glutamatergic receptor subunits, suggesting that the glutamate released by CR cells is involved in the maturation of the circuit, in line with another study (Anstötz et al., 2022). Regardless of the specific pathways involved, this is the first study to investigate the non-cell autonomous molecular landscape of the hippocampus after premature ablation of CR cells and demonstrates that their role in postnatal development is multifaceted. Future work focused on more refined manipulation will likely reveal insights into the mechanism underlying the specific alterations reported.

The hippocampal formation matures during the first weeks of postnatal development (Alberini and Travaglia, 2017; Donato et al., 2017; Dumas, 2005), as hippocampal function slowly emerges (Wills et al., 2013). It has been proposed that the hippocampus undergoes a crucial developmental period, centered around the third postnatal week, and that its maturation is experience dependent (Alberini and Travaglia, 2017; Travaglia et al., 2016). This time interval correlates with the drastic increase in spine density, particularly of the thin type, that we observed in control animals. Thin spines are subject to a faster turnover and are considered less mature than mushroom spines, which are very stable and can persist for months (Holtmaat et al., 2005; Majewska et al., 2006; Zuo et al., 2005). Thin and mushroom spines are considered to be more involved in learning and memory processes, respectively (Bourne and Harris, 2007; Kasai et al., 2003). Moreover, the density of thin spines has been suggested to indicate the plastic capacity of the neuronal network (Berry and Nedivi, 2017). By controlling thin spine density during postnatal development, CR cells might be involved in regulating mechanisms that are crucial for hippocampus-dependent learning and plasticity.

Pharmacological ablation of CR cells in organotypic slice cultures prevents the ingrowth of entorhinal cortex fibers into the hippocampus (Del Río et al., 1997). As the axon of CR cells extends to several areas of the hippocampal region, reaching the entorhinal cortex (Anstötz et al., 2016; Deng et al., 2007; Supèr et al., 1998), it has been proposed that, at the earliest developmental stages, fibers from the entorhinal cortex could use CR cells axons as a scaffold to reach the hippocampus, where they will transiently contact CR cells (Supèr et al., 1998). Here, we can contribute to the suggested role of CR cells as regulators of the development of the entorhinal-hippocampal circuit postnatally. In fact, the absence of hippocampal CR cells resulted in a reduction of Lphn2. Lphn2 has been proposed to function as a postsynaptic guidance molecule for the hippocampal network, with peak expression at P8 during development (Pederick et al., 2021), and as an adhesion molecule for the maintenance of these connections in the adult (Anderson et al., 2017). The observed changes on Lphn2 suggest that CR cells also contribute to the maturation of the entorhinal-hippocampal circuit during postnatal development. Indeed, the interesting trend observed in both Lphn2 and thin spines in the SLM hints at a direct correlation between spine density and the expression levels of this gene/protein: at the early time points analyzed, we found higher levels of Lphn2 and a higher density of thin spines in the experimental group, whereas at later time points the levels of both Lphn2 and spine density were decreased. Further experiments in the future will be needed to understand the causative relationship between these two observations.

A potential concern could be that the observed changes in spine density are caused by the loss of the CR cell to CA1 pyramidal cell connection, especially as CR cells have a postsynaptic target in these cells (Quattrocolo and Maccaferri, 2014). However, this is unlikely because optogenetic stimulation of CR cells elicited responses in only 14% of recorded pyramidal cells, suggesting a rather sparse connectivity between these cell types (Quattrocolo and Maccaferri, 2014). In addition, electron microscopy showed that 85% of the connections made by CR cells are on dendritic shafts, and only the remaining 15% are on spines, indicating a strong bias towards CR cells targeting interneurons (Anstötz et al., 2016). Based on the electrophysiological and electron microscopy experiments, we are confident that the CR to pyramidal cells connectivity is too small to have the impact we see in our morphological analysis. In addition, we have observed a 45% reduction in spine density in the SR, a layer in which there are no CR cell somata or axons. In fact, in the SR we find mainly the Schaffer collaterals – the fibers from CA3 pyramidal cells (Witter, 2018). As we did not observe an abnormal expression of Lphn2 in other hippocampal layers, and there was a reduction in spine numbers in both SLM and SR at later timepoints, it is unlikely that the ablation of CR cells leads to mistargeting of EC fibers, at least not at the level of the target layer. The most simplistic explanation for our observations is that of a general reduction in the number of contacts between axons from EC and CA3 to CA1 pyramidal cells. Altogether, these data suggest that CR cells have an important role in the establishment of the different hippocampal microcircuits. In the future, it will be important to reveal the molecular pathways regulating the maturation of the layer-specific connectivity to understand how the observed changes impact the function of the different areas of the hippocampal formation. In conclusion, our work reveals a crucial role for CR cells in the postnatal maturation of the hippocampal circuit, highlighting the importance of this transient cell population in the region, and raising new and interesting questions on their impact on hippocampal function.

## MATERIALS AND METHODS

### Animals

Male mice belonging to the Pde1c-Cre transgenic mouse line [B6.FVB(Cg)-Tg(Pde1c-cre)IT146Gsat/Mmucd, MMRRC 030708] (Osheroff and Hatten, 2009) were bred with wild-type females (C57BL/6JBomTac, Taconic). Both male and female mice originating from this cross were used for experiments in this study.

All experiments were conducted in compliance with protocols approved by the Norwegian Food Safety Authorities and European Directive 2010/63/EU (FOTS ID 24847). All mice were housed in enriched environment cages in an inverted 12 h light/dark cycle with food and water ad libitum. Pups were separated from the mother at postnatal day 21 (P21).

### Viral injection procedure

All pups were subjected to viral injection at P0. These were bilateral injections with a recombinant adeno-associated virus (pAAV-mCherry-flex-dtA Addgene plasmid 58536) (Wu et al., 2014) expressing Cre-dependent diphtheria toxin A fragment (AAV-DTA). Animals were anesthetized with isoflurane (3%) and head fixed in a stereotaxic frame (Koppf) with a custom-made adaptor. The skin was stretched using standard lab tape with a diamond cutout. Injection coordinates were calculated from *lambda* (AP, +0.8 mm; ML, ±1.2 mm; Z, −1.22 mm) in each mouse. A virus-filled glass pipette (Drummond Scientific Company) was attached to a Nanoject III injector (Drummond Scientific Company) for the injections (48.4 nl, divided into four injections of 12.1 nl volume, rate 10 nl/s, with 2 s delay).

Injected Cre^+^ animals (Pde1c-Cre^+/−^;DTA) were considered to be the experimental group and their Cre^−^ littermates (Pde1c-Cre^−/−^;DTA) were considered as controls. Only animals that had cells expressing mCherry in the inner blade of the dentate gyrus were used for experiments as this indicated viral spread to most of the hippocampus.

### Euthanasia

For euthanasia, all animals were first anesthetized with isoflurane before being euthanized with a lethal intraperitoneal injection of pentobarbital (100 mg/kg).

### Immunohistochemistry

After euthanasia, the animals were transcardially perfused first with phosphate-buffered saline (PBS) followed by a 4% paraformaldehyde (PFA) solution in PBS (14 ml at a rate of 3.7 ml/min). Brains were harvested and postfixed 3 h in PFA. They were then moved to a 15% sucrose solution overnight (stored at 4°C) and then to a 30% sucrose solution for an additional overnight incubation. Fixed brains were coronally sectioned on a freezing microtome (ThermoScientific) at a 50 µm and stored in cryoprotective solution (40% PBS, 30% glycerol and 30% ethylene glycol).

For immunohistochemistry, sections were rinsed in PBS for 10 min and subsequentially blocked for 30 min in a blocking solution containing 10% normal donkey serum (Jackson ImmunoResearch,) and 0.1% Triton X-100 (Merk) dissolved in PBS. Antigen retrieval was performed once, only for staining of latrophilin 2 (Lphn2), when sections were heated in a basic antigen retrieval reagent (sodium citrate at pH 8.5) at 75°C for 10 min before blocking. Sections were then incubated with primary antibodies diluted in an incubating solution containing 1% normal donkey serum and 0.1% Triton X-100 dissolved in PBS, overnight for three nights at 4°C with gentle shaking. Primary antibodies were as follows: rat anti-RFP to amplify the mCherry signal, dilution 1:1000 (Chromotek, 5F8); rabbit anti-p73, dilution 1:500 (Abcam, ab40658); mouse anti-reelin, dilution 1:500 (Abcam, ab78540); and rabbit anti-Lphn2, dilution 1:1000 (Novus Biologicals, NBP2-58704). The secondary antibody staining (directly conjugated Alexa fluorophores, 1:500 diluted in incubating solution) followed after 3×1 h rinsing with PBS overnight at 4°C. Secondary antibodies were as follows: donkey anti-mouse Alexa 488 (Invitrogen, A-21202); donkey anti-rabbit Alexa 488 (Abcam, ab150073); donkey anti-rat Alexa 594 (Abcam, ab150154); and donkey anti-rabbit Alexa 647 (Abcam, ab150154). The next day, sections were rinsed three times for 10 min each in PBS, then incubated in PBS with 1:200 Nissl (Invitrogen, N21479) for 1 h. Sections were then rinsed in PBS-0.1% Triton-X for 10 min followed by a 1 h rinse in PBS. Finally, sections were mounted on SuperFrost glass slides (Thermo Fisher Scientific) in a Petri dish with PBS, briefly rinsed with distilled water, coverslipped with mounting medium (Fluoromount-G, ThermoFisher), then sealed with nail polish and stored at 4°C.

### Confocal imaging and cell counts

A LSM880 Zeiss Confocal Microscope was used to acquire tiled *z*-stack images of the hippocampal fissure and molecular layer of the dentate gyrus using a Zeiss plan apochromat 20×/numerical aperture 0.8 objective. Four coronal sections from each animal were chosen for imaging. Sections were 150 μm apart, demonstrating a minimum viral spread of 600 μm.

Confocal imaging files containing the dorsal hippocampus were uploaded in Neurolucida (Micro Bright Field Bioscience) for analysis. A contour delineating the stratum lacunosum-moleculare and the molecular layer of the inner blade of the dentate gyrus was created. Symbols for each signal were used to count cells labeled by different markers. To quantify double or triple labeled cells, a different marker was used for each combination of signals. We counted all cells contained within the contours. As non-consecutive slices were counted, overcounting the *z*-axis was not relevant and no correction was applied. Quantification of the number of markers in the contour was carried out in Neurolucida Explorer (Micro Bright Field Bioscience) and exported in excel files. To assess the specificity of the Pde1c-cre mouse line, the number of cells was used to calculate the ratio (Fig. 1B). To assess the effect of the AAV-DTA virus, the same delineations were made but only cells immunolabeled with p73 were counted. The density of neurons labeled by a marker was measured as the number of labeled neurons in a contour divided by the area included in the contour (cells/mm^2^).

### Single cell imaging

Confocal images were acquired with a Plan-Apochromat 63×/1.4NA oil DIC M27 objective. Pixel size was set to 0.07 µm in X and Y, and 0.2 µm in Z. This oversampling was carried out to accommodate the Nyquist sampling rate, ensuring optimal resolution. Pixel dwell was set to the shortest duration for every acquisition (40±10 µs), with an average of 2, and the images were captured with a bit depth of 12 bit. Acquisition was carried out over one to six cells at a time, looking for somata that were positioned at approximately the same Z-level. A stack of 4.5±0.3 µm was performed to capture as much of the somata as possible. All four channels were put on different tracks to ensure as little crosstalk as possible.

Images were deconvoluted using Huygens deconvolution software (v22.10, Scientific Volume Imaging, The Netherlands, http://svi.nl) with a theoretical point spread function. The background was automatically estimated and double checked for error by the operator. The images were deconvoluted with 100 iterations, a quality threshold of 0.001 and an acuity of 0.0.

After deconvolution, Fiji/ImageJ (V1.154d, National Institute of Health, USA, https://imagej.net/) was used to create a maximum intensity projection. All channels were split and a region of interest was drawn around the soma of the cells based off their respective counterstain (CR cells – tdTomato^+^ and Reelin^+^, Interneurons – Reelin^+^ and tdTomato^−^, Pyramidal cells – NeuN^+^). An average pixel intensity was obtained from the Lphn2 channel inside the soma-ROI, giving an average pixel intensity for Lphn2 for every imaged cell. Total cell number selected for analysis equated to 90 cells collected from three animals (10 cells per cell group, from two different nonconsecutive sections).

### Densitometric analysis

Lphn-2 and mCherry antibody conjugated *z*-stack confocal images were acquired from seven P30 mice (Pde1c-Cre^−/−^;DTA *n*=3, Pde1c-Cre^+/−^;DTA *n*=4) from three separate coronal slices of dorsal hippocampus in each sample. To assess the signal distribution of both Lphn2 and mCherry, image stacks were processed and analyzed in FIJI/ImageJ. Images stacks were compressed to *z*-projections PNG files, a standard rectangular region of interest (ROI) was drawn starting from the stratum oriens and extending to the boundary of the molecular layer of the outer blade of the dentate gyrus for a total length of 1250 µm and width of 100 µm. Signal values from respective proteins were quantified using the densitometry function. Briefly, the densitometry function averages signal intensities within a singular discreet distance along the short axis of the rectangular ROI such that each pixel with the same *x*-axis location will be averaged together and repeated over the long axis of the rectangle. All three coronal slices were averaged for each of the individual slices within their respective protein groups and then the signal values for Lphn2 were normalized to the signal values for mCherry. The resulting curves were compared between experimental groups.

### Confocal imaging and whole-cell reconstruction

Cells were filled with biocytin during whole-cell recording and then fixed overnight in 4% paraformaldehyde in 0.1 M PBS at 4°C. Fluorescent and biocytin signals were detected with the following procedure: sections were rinsed in PBS three times for 5-10 min for each rinse, and then blocked for an hour in a blocking solution containing 10% normal donkey serum with 0.5% Triton X-100. Slices were then moved into incubating solution with 0.5% Triton X-100 with rat anti-RFP antibody for mCherry staining (1:500, Chromoteck) over night for 3 nights at 4°C with gentle shaking. Slices were then rinsed three times for 1 h in PBS, then moved into incubating solution with 0.5% Triton X-100 with streptavidin-conjugated Alexa Fluor 488 (1:500, Invitrogen) and donkey anti-rat secondary antibodies conjugated to Alexa Fluor 594 (1:500, Invitrogen) and incubated overnight at 4°C with gentle shaking. The following day, slices were rinsed five times for 10 min each in PBS and then mounted onto SuperFrost glass slides (Thermo Fisher Scientific).

Images for whole-cell reconstructions were acquired from 300 µm sections using a Plan-Apochromat 20×/NA 0.8 objective. The frame size was set to 2048×2048 pixels and 0.6× digital zoom, resulting in a pixel size of 0.35 μm in X and Y, and a Z-step of 0.85 μm. (pinhole size of ∼1 Airy Unit, 8-bit depth, ∼0.5 μsec pixel dwell, an averaging of 2 and unidirectional scan direction). Only one channel was acquired, excited by an Argon 488 nm laser, emission filter set in the range of 490-550 nm.

Whole cells were reconstructed using Neurolucida 360 with user guided tracing. Dendrites were separated into two main groups: all dendrites extending superficially from the soma were defined as apical and all dendrites extending deep were defined as basal. The axon could be identified in most images, and was reconstructed and classified to exclude it from the dendritic analysis. Additionally, in every image, delineations of hippocampal layers were drawn by eye, based on autofluorescence. All data acquisition was performed by an experimenter who was unaware of the mouse genotype.

### Whole-cell morphological analysis

Sholl analysis data and dendritic length were collected from the reconstructed cells using Neurolucida explorer. In the Sholl analysis, the initial ring radius was set to 10 μm and the increment in radius of subsequent rings was 10 μm. Dendritic length was calculated based on the skeletal trace through dendrites, grouped by hippocampal layer.

### Dendritic reconstruction and spine counting

Neurons were imaged using a LSM880 Zeiss Confocal Microscope with a Zeiss plan apochromat 63×/numerical aperture 1.4 oil immersion objective on thick sections (300 µm). The frame size was set to 1024×1024 pixels, 4.0× digital zoom, resulting in a pixel size of 0.03 μm in X and Y, and a Z-step of 0.4 μm (pinhole size of ∼1 AU, 12-bit depth, ∼1.02 μs pixel dwell, an averaging of 4 and bidirectional scan direction). Only one channel was acquired, excited by an Argon 488 nm laser, with the emission filter set in the range of 490-550 nm.

Each neuron was imaged as part of a threefold experiment: imaging three dendritic segments in the SLM, the stratum radiatum (SR) and finally the stratum oriens (SO). Dendritic segments were chosen based on the same criteria: each segment is part of a terminal dendrite defined as the last bifurcation of the dendritic shaft. Cells without apical dendrites reaching the SLM were excluded from the experiment. This resulted in a distribution of *n* cells and *N* animals per postnatal day: P22 (*n*_Pde1c−Cre−/−;DTA_= 1, *N*=1; *n*_Pde1c−Cre+/−;DTA_= 4, *N*=1), P23 (*n*_Pde1c−Cre−/−;DTA_=1, *N*=1; *n*_Pde1c−Cre+/−;DTA_=2, *N*=1), P25 (*n*_Pde1c−Cre−/−;DTA_=1, *N*=1), P26 (*n*_Pde1c−Cre−/−;DTA_=1, *N*=1, *n*_Pde1c−Cre+/−;DTA_=1, *N*=1), P28 (*n*_Pde1c−Cre+/−;DTA_=1, *N*=1), P29 (*n*_Pde1c−Cre−/−;DTA_=1, *N*=1, *n*_Pde1c−Cre+/−;DTA_=4, *N*=2), P30 (*n*_Pde1c−Cre−/−;DTA_=2, *N*=1), P32 (*n*_Pde1c−Cre−/−;DTA_=2, *N*=1), P34 (*n*_Pde1c−Cre−/−;DTA_=3, *N*=2, *n*_Pde1c−Cre+/−;DTA_=1, *N*=1) and P35 (*n*_Pde1c−Cre−/−;DTA_=1, *N*=1). All data acquisition and analysis were performed by an experimenter who was unaware of the mouse genotype.

The image was deconvolved by Huygens Deconvolution Software using Express Mode with a Classic Maximum Likelihood Estimation protocol with a theoretical point spread function (PSF) generated by the Huygens software based on the properties of the immersion objective. Dendritic shafts were reconstructed using Neurolucida Software (Micro Bright Field Bioscience) by means of user-guided tracing with directional kernels. Spines were tagged using unique markers to classify spine subtypes. This spine classification was based on established criteria: thin spines had a neck length greater than their head diameter; mushroom spines had a much greater diameter of their head compared with their neck; and whereas stubby spines had no identifiable neck (Harris et al., 1992). Spines were subsequently quantified by means of Branch Structure Analysis and Markers and Regions Analysis on Neurolucida Explorer (Micro Bright Field Bioscience).

### Whole hippocampus extraction

P15 and P30 Pde1c-Cre mice, previously injected at P0 with AAV-mCherry-flex-DTA were euthanized and decapitated. After decapitation, the brain was removed from the skull and immersed in an ice-cold buffer [320 mM sucrose and 10 mM Hepes (pH=7.4)]. The hippocampi were then dissected and checked for injection reporter using a Stereo Microscope Fluorescent Adapter with a Green lamp (Nightsea). Samples were included only if an intense red fluorescence was detected in the dorsal hippocampus and only the red fluorescent part was dissected.

### Microdissection of CA1 SLM

P15 and P30 Pde1c-Cre mice, previously injected at P0 with AAV-mCherry-flex-DTA, were euthanized and decapitated. After decapitation, the brain was removed from the skull and immersed in chilled sucrose ACSF with the following composition (mM): NaCl 87, KCl 2.5, NaH_2_PO_4_ 1.25, NaHCO_3_ 26, sucrose 75, glucose 10, MgCl_2_ 7, CaCl_2_ 0.5, saturated with 95% O_2_ and 5% CO_2_ (pH 7.4). Coronal sections (500 µm) were cut using a vibrating microtome (VT1000S, Leica). Slices containing the dorsal hippocampus were quickly moved to a petri dish containing chilled sucrose ACSF and correct targeting of the viral injection was assessed with the use of a Stereo Microscope Fluorescent Adapter with a Green lamp (Nightsea). Only slices in which bright red fluorescence was visible in the entire hippocampus were used for the microdissection. A V8 Discovery Stereoscope (Zeiss) was used to guide the microdissection of the CA1 SLM. The CA1 SLM was finitely dissected using a Barkan microknife (FST) and Dumont number 5 forceps (FST). Tissue was taken from two subsequent slice sections from both hemispheres and was kept on ice until RNA extraction.

### RNA extraction

Whole hippocampus or microdissected CA1 SLM was processed for RNA extraction. Tissue was homogenized and processed using either a Qiagen RNAEasy (Qiagen) or a Nucleospin RNA extraction kit (Machery-Nagel). Isolated RNA from whole hippocampus was analyzed using bulk mRNA Seq, whereas isolated RNA from CA1 SLM was analyzed using qRT-PCR.

### Bulk mRNA sequencing

RNA from snap-frozen injected whole hippocampus tissue was quality controlled using Agilent 2100 Bioanalyzer and RNA quantity was detected by Qubit fluorometric quantitation. Samples were sequenced at a minimum RIN value of 8.0. Libraries were performed using the Illumina Stranded mRNA Prep, Ligation kit. High-throughput sequencing was performed using an NS500HO flowcell (Illumina) for an average read of ∼16×10^6^ reads. Fastq files were assessed using bcl2fastq/QC report. Sequencing was performed at the Genomics Core Facility at NTNU.

### Transcriptome analysis

Raw read files were assessed for quality and then pseudoaligned to GRCm39 *Mus musculus* using kallisto (Bray et al., 2016). Aligned transcriptomic data processing and analysis was executed in RStudio (RStudio IDE 2021.09.0 Build 351) using tximport (Soneson et al., 2015), ensembldb (Rainer et al., 2019), edgeR (Robinson et al., 2010), matrixStats, limma (Ritchie et al., 2015), GSEABase (Morgan et al., 2022), GSVA (Hänzelmann et al., 2013), ClusterProfiler (Koopmans et al., 2019), enrichplot (Yu, 2022) and SynGO (Koopmans et al., 2019). Raw reads were normalized using trimmed means of M-values (TMM) and genes were disregarded if the expression was less than one in at least four samples. Significant gene expression was determined using threshold values of LogFC≥2 and Benjamini-Hochberg adjusted *P*-values≤0.05. SynGO analysis was performed using the top 200 genes (<1% of kept genes) in a ranked list of adjusted *P*-values. All samples were analyzed from P15 Pde1c-Cre^−/−^;DTA (*n*=4), P15 Pde1c-Cre^+/−^;DTA (*n*=4), P30 Pde1c-Cre^−/−^;DTA (*n*=4) and P30 Pde1c-Cre^+/−^;DTA (*n*=4) to control for genotype variability at each age until pairwise analyses were performed. Plots were created using cowplot and ggplot.

### qRT-PCR

RNA from microdissected CA1 SLM samples were processed using Reverse Transcriptase Core Kit 300 (Eurogentec RT-RTCK-03). Approximately 100 ng of RNA was reverse transcribed into cDNA for qRT-PCR analysis. SensiFAST SYBR mix (12 ml, BioLine BIO-98020) and cDNA (8 ml) were combined in tubes and quantified using StepOne*Plus* (Thermo Fisher). Primers were designed using NIH Primer Blast with the most recent genome sequencing of *Mus musculus*. All primers were designed with predicted amplicon product ranges of 50-150 bp. Take-off cycles thresholds (C_T_) for each gene were normalized to the respective *Gapdh* take-off-cycle of each gene.

### Primers

Primers used for each gene were as follows: *Gapdh* forward, CTGCACCACCAACTGCTTAG; *Gapdh* reverse, ATCCACAGTCTTCTGGGTGG; *Lphn2* forward, TCTGGAACAGAGGGCGAAG; *Lphn2* reverse, TAGCCAGAAGGAATATGGAAGAGT; *Igsf8* forward, CAGGAGTCGCCCTAGTTACC; *Igsf8* reverse, TGGCTGGAAGACAGTCAACA; *Neo1* forward, AAAGGCCTCCCGAAAAAGTG; *Neo1* reverse, AGCAGCGACTCGGAAGTTAT; *Robo2* forward, TCATGGGTCCCAAAACTTGC; *Robo2* reverse, TGCAGGCACTGATGGTAGAA.

### Sample preparation for liquid chromatography tandem mass spectrometry (LC-MS/MS)

Tissue samples were microdissected as described above. Total protein extractions were performed with the Total RNA and Protein Isolation Kit from Macherey-Nagel following the manufacturer's instructions. Protein concentrations of all samples were detected with the Protein Quantification Assay (Macherey-Nagel) using BSA as standard and maximal 1 µg of total protein extract processed for LC-MS/MS analyses. LC-MS/MS sample preparation and analyses were performed using the Proteomics and Modomics Experimental Core Facility (PROMEC, NTNU). In brief, tryptic peptide samples were generated by in-gel digestion. Protein samples were run for 200 V for 6 min using Invitrogen Bolt Bis-Tris Plus gel with MOPS running buffer (ThermoFisher). Proteins were visualized using Readyblue Protein Gel Stain (Sigma). The gel lanes were cut out and proteins were digested using trypsin. The gel pieces were distained using repeatedly 5 min washes with 50 mM NH_4_HCO_3_/50% (v/v) CH_3_CN until visible Coomassie were removed. The gel pieces were dehydrated by adding 500 µl 100% CH_3_CN until the gel pieces shrunk and became white. The gel pieces were rehydrated using 10 mM DTT in 50 mM NH_4_HCO_3_ at 60°C for 30 min. The samples were cooled down to room temperature and 5 mM iodoacetamide in 50 mM NH_4_HCO_3_ was added and incubated in dark for 30 min at room temperature. After reduction and alkylation, the solution was removed and the gel pieces again dehydrated as before. Trypsin 12.5 ng/µl in 50 mM NH_4_HCO_3_ was added until the gel pieces were rehydrated. Finally, 50 mM NH_4_HCO_3_ was added to keep the gel pieces wet during incubation at 37°C overnight. After digestion, the samples were dried using a speedvac. Peptides were resuspended in 100 µl 0.1% (v/v) trifluoroacetic acid and desalted using Oasis-HLB 96 well C18 SPE plates. After elution of peptides from C18, the samples were dried in a speedvac, further dissolved in 15 µl 0.1% (v/v) formic acid and LC-MS/MS was performed on a timsTOF Pro (Bruker Daltonics) connected to a nanoElute (Bruker Daltonics) HPLC. Peptide separation was carried out using an Aurora (75 µm×25 cm) column with running buffers A [0.1% (v/v) formic acid] and B [0.1% (v/v) formic acid in acetonitrile] with a gradient from 0% B to 40% B for 80 min. The timsTOF instrument was operated in the DDA PASEF mode with 10 PASEF scans per acquisition cycle, and accumulation and ramp times of 100 ms each. The ‘target value’ was set to 20,000 and dynamic exclusion was activated and set to 0.4 min. The quadruple isolation width was set to 2 Th for m/z <700 and to 3 Th for m/z>800.

### Proteomic data analyses

LC-MS/MS raw data were interpreted with MaxQuant 2.2.0.0 software (Cox and Mann, 2008) using the mouse proteome, including isoforms from Uniprot (downloaded: 19.07.2022) as a reference dataset for protein identification. Proteins were identified with one unique peptide and a false discovery rate (FDR) of 0.01. Search parameters were set as follows: Trypsin/P as a digestion enzyme; two missed cleavages were allowed; six amino acids were set as minimum peptide length; oxidation (M), acetyl (protein N-term) and deamidation (NQ) were included as variable modifications. Label-free quantification (LFQ) was performed with match between runs enabled using MaxQuant standard settings with an alignment time window of 25 min. MaxQuant results were quality controlled with the PTXQC package (Bielow et al., 2016). Synaptic protein groups were identified by using SynGo (Koopmans et al., 2019) analysis.

### Statistical analysis

For cells, dendrites and spine quantification, MATLAB_R2019b (The MathWorks Inc) was used to extract all statistical measures. Number of cells per µm^2^ area was converted to mm^2^ and then averaged across four sections of each brain. Brain averages of CR cells densities per mm^2^ were statistically compared through a two-sample *t*-test assuming unequal variance.

Two different statistical tests were used for significance testing of dendritic analyses. For the Sholl analysis, apical and basal dendritic arborization were conducted separately. To avoid multiple comparisons, we simply compared the area under the curve that was generated for both the apical and basal dendrites. Area under the curve was calculated using the trapezoidal rule with a step size corresponding to each Sholl ring, and subsequently compared using a Mann–Whitney-*U-*test. For dendritic length analysis, an independent-samples *t*-test was used again, comparing the experimental and the control group for each hippocampal layer. Both analyses were performed using IBM SPSS Statistics v.27.0 (IBM).

Raw dendritic spine data was standardized by dendritic length to correct for variations in dendritic branching and uneven sample numbers between groups. As such, spine density for each dendritic segment was given by the spine quantity divided by branch length (µm). This equation was applied to the total spine density (comprising all spine markers) and subcategories that included unique markers for filopodia, thin, stubby and mushroom spines. A simple linear regression model was applied to quantify age-related effects in the data across all hippocampal layers using postnatal age as the predictor variable. To analyze a difference directly between the regression models generated for each group, we applied an ANCOVA (analysis of covariance). This allowed us to compare the intersections of the regression slopes, as well as the slopes themselves.

Statistical analysis of qRT-PCR data was performed using Graphpad Prism v8.0.2. ΔCt values were compared between groups for each gene using mixed-effects multiple comparison analysis corrected using FDR in two-stage step-up method of Benjamini, Krieger and Yekutieli approach with a Q threshold of 1%. This analysis was chosen to adjust for the number of samples, number of genes and number of groups analyzed. Statistical significance is reported as the adjusted *P*-value.

To statistically analyze the Lphn2 signal density curves between P30 groups in Pde1c-Cre^−/−^;DTA (*n*=3) and Pde1c-Cre^+/−^;DTA (*n*=4) groups, the curve for each sample was used to calculate the average experimental curve and the resulting curves were analyzed with three statistical tests in GraphPad Prism 9. First, the curves were analyzed using Deming Regression testing to determine whether the slopes were non-zero, then followed by Wilcoxon matched pairs between individual data points to test variance between signals along each discreet short-axis distance point, and finally the area under the receiver operating characteristic (ROC) curve was analyzed to determine cumulative signal changes between experimental groups.

Proteomic results generated with MaxQuant were processed using MSstats (Choi et al., 2014) running on the Galaxy Europe server (usegalaxy.eu; The Galaxy, 2022). For the data analyses, the following biological replicates were considered: P15 Pde1c-Cre^−/−^;DTA (*n*=6), P15 Pde1c-Cre^+/−^;DTA (*n*=4), P30 Pde1c-Cre^−/−^;DTA (*n*=2) and P30 Pde1c-Cre^+/−^;DTA (*n*=7).

## Supplementary Material


