# Supplementary material for: Postnatal persistence of hippocampal Cajal-Retzius cells has a crucial role in the establishment of the hippocampal circuit
Source: Development. 2024 Jan 9;151(1):dev202236. doi: 10.1242/dev.202236 (PMC10820737; doi:10.1242/dev.202236)
Supplement: Supplementary file 1 [file develop-151-202236_review_history.pdf]

# Postnatal persistence of hippocampal Cajal-Retzius cells has a crucial role in the establishment of the hippocampal circuit

Ingvild Lynneberg Glærum, Keagan Dunville, Kristian Moan, Maike Krause, Nicola Pietro Montaldo, Hinako Kirikae, Maximiliano Jose Nigro, Pål Sætrom, Barbara van Loon and Giulia Quattrocolo

DOI: 10.1242/dev.202236

**Editor**: Francois Guillemot

**Review timeline**

| | |
|---|---|
| Original submission: | 4 August 2023 |
| Editorial decision: | 4 September 2023 |
| First revision received: | 10 November 2023 |
| Accepted: | 27 November 2023 |

---

**Original submission**

First decision letter

MS ID#: DEVELOP/2023/202236

MS TITLE: Postnatal persistence of hippocampal Cajal-Retzius cells has a critical role in the establishment of the hippocampal circuit

AUTHORS: Ingvild Lynneberg Glærum, Keagan Dunville, Kristian Moan, Maike Krause, Nicola Pietro Montaldo, Hinako Kirikae, Maximiliano Jose Nigro, Pål Sætrom, Barbara van Loon, and Giulia Quattrocolo

I have now received all the referees' reports on the above manuscript, and have reached a decision. The referees' comments are appended below, or you can access them online: please go to BenchPress and click on the 'Manuscripts with Decisions' queue in the Author Area.

As you will see, the referees express considerable interest in your work, but have some significant criticisms and recommend a substantial revision of your manuscript before we can consider publication. If you are able to revise the manuscript along the lines suggested, which may involve further experiments, I will be happy receive a revised version of the manuscript. Your revised paper will be re-reviewed by one or more of the original referees, and acceptance of your manuscript will depend on your addressing satisfactorily the reviewers' major concerns. Please also note that Development will normally permit only one round of major revision. If it would be helpful, you are welcome to contact us to discuss your revision in greater detail. Please send us a point-by-point response indicating your plans for addressing the referees' comments, and we will look over this and provide further guidance.

Please attend to all of the reviewers' comments and ensure that you clearly highlight all changes made in the revised manuscript. Please avoid using 'Tracked changes' in Word files as these are lost in PDF conversion. I should be grateful if you would also provide a point-by-point response detailing how you have dealt with the points raised by the reviewers in the 'Response to Reviewers' box. If you do not agree with any of their criticisms or suggestions please explain clearly why this is so.

Reviewer 1

© 2024. Published by The Company of Biologists under the terms of the Creative Commons Attribution License (https://creativecommons.org/licenses/by/4.0/).

*Advance summary and potential significance to field*

This is a clear and careful study that uses genetic tools to probe the role of the CR cells that persist postnatally in the hippocampus. This is an interesting question, as CR cells are known to fundamentally shape cortical architecture during development, but predominantly disappear before birth. Their persistence in the hippocampus during normal development provides an interesting case to compare their distinct or overlapping roles postnatally. The work is significant in that it shows specific ablation of CR cells postnatally impacts hippocampal development, and thereby reveals a developmental role for this cell type specific to the hippocampus. This is an advance in the field's understanding of the roles of CR cells, and is particularly significance because of the postnatal timing of this normal developmental role.

*Comments for the author*

1. In the discussion the authors touch upon the possibility that some of the impacts of CR ablation on hippocampal synaptogenesis might be due to glutamatergic release from CR cells. This is an attractive hypothesis, as a highly parsimonious option would be that the “lost” synapses and dendritic complexity in the Cre+ condition is due solely to the loss of pre-synaptic connectivity from absent CR cells themselves (and not necessarily any other cell type). Further attention to this point may help to narrow down the candidate mechanisms driving this effect.
For example, an estimation of the degree of connectivity from CR cells to CA1 cells (either from the literature or otherwise) and comparing this to the degree of synaptic loss may be informative. If this isn’t possible, further speculation on this point may help future studies to narrow down candidate mechanisms.

2. Along the same lines as the point above and following a similar rationale, it would be of interest to know whether the reduction of Lphn2 in Cre+ animals shown in Figure 8C is specific to the CR cells themselves, or also lost in non-CR cells.
This could be achieved by co-labelling with P75 or reelin and Lphn2, where the fluorescence intensity of Lphn2 is compared within P75/reelin positive and negative cells in both conditions.

3. For Figure 2C – was there correction for multiple comparisons applied? If not why?

4. For Figure 4 – It would seem more logical to present the p values for the ANCOVA tests on each graph as well as or even instead of the p values for the regression model of each condition in isolation. At the moment it’s hard to quickly compare the statistical differences between conditions from the figure.

<u>Reviewer 2</u>

*Advance summary and potential significance to field*

The authors make use of an elegant experimental paradigm to get rid of a crucial developmental cell population, CR cells, and investigate their role in circuit formation. There is a clear experimental pipeline that details the sequence of findings that bring to the final proposal. The authors are knowledgeable in this matter and their results/interpretations are mostly well supported (except for the cases stated below). I truly acknowledge the value of the discussion section, which truly compares this novel data to many recent and classic articles in the field. Altogether, I am convinced this article would bring many novel concepts and results to the field, and would be greatly appreciated and referenced, not only by researchers working directly on hippocampus or Cajal-Retzius, but also in general brain and cortical development as well as circuit formation. Cajal-Retzius cells are making much more than originally expected.

*Comments for the author*

In conclusion, this is a good piece of research that conducts experimentation in a logic fashion. The interpretation of their results is adequate, and their conclusion are mostly well supported by their

© 2024. Published by The Company of Biologists under the terms of the Creative Commons Attribution License (https://creativecommons.org/licenses/by/4.0/).

data. There are only some comments and suggestions that I would like the authors to address prior publication.

• My main concern is the direct relation of the loss of CR cells to the malformed hippocampal circuitry. The authors unambiguously identify that in absence of CR cells fewer synapses are made on CA1 neurons at the SLM level. What is not clear to me is the neuronal origin of these lacking synapsis in the SLM. Surely CR cells may contact CA1 cells at this layer, and it is obvious that when CR cells are not present, their synapses are gone. If the origin of the missing synapsis is primarily CR cells, their loss would not be affecting hippocampal circuitry, but only a dull, direct connection CR-CA1. The authors discuss that the origin of these synapsis are entorhinal neurons, but there is no evidence for this, neither coming from the literature nor from direct experimentation. In my opinion, the entorhinal origin of SLM synapsis must be proven in a more convincing way, by either direct tracing or comprehensive literature on the synapsis coming from CR cells. Until then, the lack of CR cells may seem to be affecting only their own missing connections.

• Are there any hippocampal populations not directly connected to CR cells? If so, I would suggest to quickly check whether their synaptogenesis is somehow altered as well.

• Some relevant experiments are missing the comparison to the total control, which is the uninfected hippocampus. Because maybe the simple viral infection without CR loss may affect somehow the circuitry or even the survival of some CR cells. Is there any difference between total control and infected animals in the number of CR cells and their marker expression (Fig. 1)? And in layer-specific dendritic spine density of CA1 pyramidal cells (Fig. 3 and 4)?

• The selection of markers of CR cells tested in fig. 5C is based in an article (Jimenez and Moreno, 2021) which is likely a bad choice. This article studies non-mammalian animals, on which the existence of CR cells is still under debate. There are plenty of great articles based on mammalian CR cells from which to extract a list of actual markers. Those on the list in Fig. 5C may be (or not) the same.

• In fig. 8C there is a clear change in the expression of Lphn2 at the hilus level and its surroundings. Could the authors mention this in the article and speculate on its origin and consequences?

• CR cells are born to die early in postnatal development, in great numbers in the cortex. It is not clear at what postnatal age post-infection CR cells die due to this experimental paradigm. It is relevant because they may die early, and affect circuit formation from very early on: changes could be there, visible, from very early after infection. Or they may take some days to die, and so the aberrant formation of the circuit could be a late event. I suggest the authors to briefly assess CR cell loss 3 and 7 days after infection, to help draw these conclusions.

<u>Reviewer 3</u>

*Advance summary and potential significance to field*

In the present study Lynneberg Glaerum and collegues induced premature death of hippocampal Cajal-Retzius cells in the early postnatal mouse hippocampus by viral vector injection. They characterized morphological changes in dendrite complexity as well as spine density of neurons of CA1 pyramidal neurons in these mice. Moreover, they characterized changes in gene regulatory networks, more specifically, they found that expression of Latrophilin-2 was significantly changed. The study has the potential to provide an important advance to the field if morphological circuit alterations of specific fiber projections could be shown by the authors.

*Comments for the author*

While a critical role for Cajal-Retzius cells in the establishment of the hippocampal circuit is proposed in the title of the study, unfortunately no morphological evidence for changed or developmentally challenged fiber projections is shown. Presenting reduced dendritic complexity is not sufficient for this claim, since this is also observed in (naturally or induced) reelin deficient mice, which nonetheless have largely normal circuits in hippocampus and neocortex. Thus, showing an altered hippocampal circuit or, in particular, an eeffect on the entorhino-hippocampal projection, would largely improve the study. Of note, the authors cite the Del Rio et al. (1997) who showed reduced ingrowth of entorhinal fibers in the hippocampus after pharmacological ablation of Cajal-Retzius cells in a co-culture system (see Discussion, line 496-97 of present manuscript). While the latter study has demonstrated this in vitro, the present in vivo model has the great potential to

© 2024. Published by The Company of Biologists under the terms of the Creative Commons Attribution License (https://creativecommons.org/licenses/by/4.0/). 

explore if these changes actually also happen in vivo after ablation of Cajal-Retzius cells. Thus, if effects on the development of fiber projections could be shown in vivo, for example by using tracing techniques, the implications would be far-reaching and therefore largely improve the study.

minor: the citation Ramon y Cajal et al is not complete in the reference list, for instance the publisher is missing.

---

**First revision**

Author response to reviewers' comments

Dear Dr. Guillemot,

Thank you for reviewing our manuscript. We are grateful for the encouragement to resubmit. We would also like to thank the reviewers for their insightful comments and their suggestions as it has made the manuscript more complete and the analyses more robust. As indicated below, we have addressed all the comments and suggestions.

Sincerely,

Giulia Quattrocolo

**Reviewer 1 Advance Summary and Potential Significance to Field:**
**This is a clear and careful study that uses genetic tools to probe the role of the CR cells that persist postnatally in the hippocampus. This is an interesting question, as CR cells are known to fundamentally shape cortical architecture during development, but predominantly disappear before birth. Their persistence in the hippocampus during normal development provides an interesting case to compare their distinct or overlapping roles postnatally. The work is significant in that it shows specific ablation of CR cells postnatally impacts hippocampal development, and thereby reveals a developmental role for this cell type specific to the hippocampus. This is an advance in the field's understanding of the roles of CR cells and is particularly significance because of the postnatal timing of this normal developmental role.**
**Reviewer 1 Comments for the Author:**

**1. In the discussion the authors touch upon the possibility that some of the impacts of CR ablation on hippocampal synaptogenesis might be due to glutamatergic release from CR cells. This is an attractive hypothesis, as a highly parsimonious option would be that the "lost" synapses and dendritic complexity in the Cre+ condition is due solely to the loss of pre-synaptic connectivity from absent CR cells themselves (and not necessarily any other cell type). Further attention to this point may help to narrow down the candidate mechanisms driving this effect. For example, an estimation of the degree of connectivity from CR cells to CA1 cells (either from the literature or otherwise) and comparing this to the degree of synaptic loss may be informative. If this isn't possible, further speculation on this point may help future studies to narrow down candidate mechanisms.**

The reviewer raised a critical point as CR cells do contact pyramidal neurons directly. However, we would argue that the effect we see is not due to this connection as we see an increased spine density at early timepoints (P22) before they are lost at later timepoints (P35) in the SLM (please see figure 4A). At these time points, we know that CR cells have been successfully ablated, as figure 1G demonstrates a drastic reduction of CR cells already at P15. We argue that the effective loss of spines towards P35 is likely not due to this direct connection. Moreover, we know from previous work that the direct connection between CR cells and pyramidal cells are rather rare, as optogenetic stimulation of CR cells elicited a response only in 14% of recorded pyramidal cells (Quattrocolo & Maccaferri, 2014). Similar experiments studying the EC to CA1 pyramidal cells connections with optogenetic simulation have demonstrated an incredibly high level of connectivity (>90%) (Li et al., 2017). In addition, electron microscopy showed that 85% of the connections made

© 2024. Published by The Company of Biologists under the terms of the Creative Commons Attribution License (https://creativecommons.org/licenses/by/4.0/). 

by CR cells are on dendritic shafts, and only the remaining 15% are on spines (Anstötz et al. 2015), arguing for a strong bias of CR cells to target interneurons. Based on both the electrophysiological experiments, and those of electron microscopy we can be fairly confident that the CR-cells to pyramidal cells connectivity is probably too small to make the impact we are seeing with our morphological analysis. In addition, it is important to consider that we see also a 45% reduction in spine density in SR, a layer in which there are no CR cells and where there are no axons from CR cells.
We have elaborated on these points in the results and discussion (Lines: 178-180 & 526- 545).

**2. Along the same lines as the point above and following a similar rationale, it would be of interest to know whether the reduction of Lphn2 in Cre+ animals shown in Figure 8C is specific to the CR cells themselves, or also lost in non-CR cells. This could be achieved by co-labelling with P75 or reelin and Lphn2, where the fluorescence intensity of Lphn2 is compared within P75/reelin positive and negative cells in both conditions.**

The reduction of Lhpn2 observed in Figure 8C is unlikely due to the absence of CR cells per se. In fact, data from RT-PCR show how at P15, a timepoint in which CR cells are gone, we have an increase of the mRNA levels of Lhpn2 (Fig.8B). Transcriptomic data available through the Allen Brain Institute also show no expression of Lhpn2 in CR cells.
https://celltypes.brain-map.org/rnaseq/mouse_ctx-hpf_10x?selectedVisualization=Heatmap&colorByFeature=Cell+Type&colorByFeatureValue= Gad1
(input Adgrl2, or see image below in which the first column, in green, represents CR cells

cells

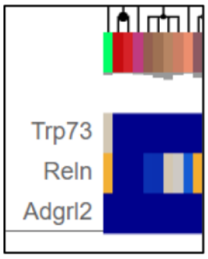


First column represents CR cells.
Value for Trp73=8.89
Value for Reln=11.61
Value for Adgrl2=0

Nevertheless, to further confirm this possibility, we performed a staining of Lhpn2 and reelin on sections of the Pde1c-Cre;flox-TdTomato mouse at P30 (figure 1 B) to check for colocalization of reelin, TdTomato (CR cells) and Lphn2. Since we expect Lphn2 to be expressed on the membrane, it will be hard to find a real colocalization using a p73 staining, which is a nuclear marker. Therefore, the use of the reporter mouse should be more conclusive, as in figure 1 (A to D) we have showed that the Pde1c-Cre;flox-TdTomato is highly specific and selective for CR cells. The results show no colocalization of Lphn2 and TdTomato in CR cells (figure 8), while we noticed expression in Pyramidal cells (as expected from Anderson et al. 2017) as well as some reelin expressing interneurons in SLM. Dendrites and axons located in SLM are known to express Lphn2 (Anderson et al., 2017; Pederick et al. 2021), explaining the strong “background” expression observed in this layer. However, notice the “gap” left by the soma of CR cells in the Lphn2 staining, indicating that these cells do not express this protein. It is particularly striking the comparison with the expression in pyramidal cells, where it is possible to identify a halo in the cytoplasm of the cells, confirming expression of Lphn2 from these cells.
We have 2 panels in Figure 8 to address this point, and we are providing few more images of cells here in support of our claim (Lines 382-393).

© 2024. Published by The Company of Biologists under the terms of the Creative Commons Attribution License (https://creativecommons.org/licenses/by/4.0/).

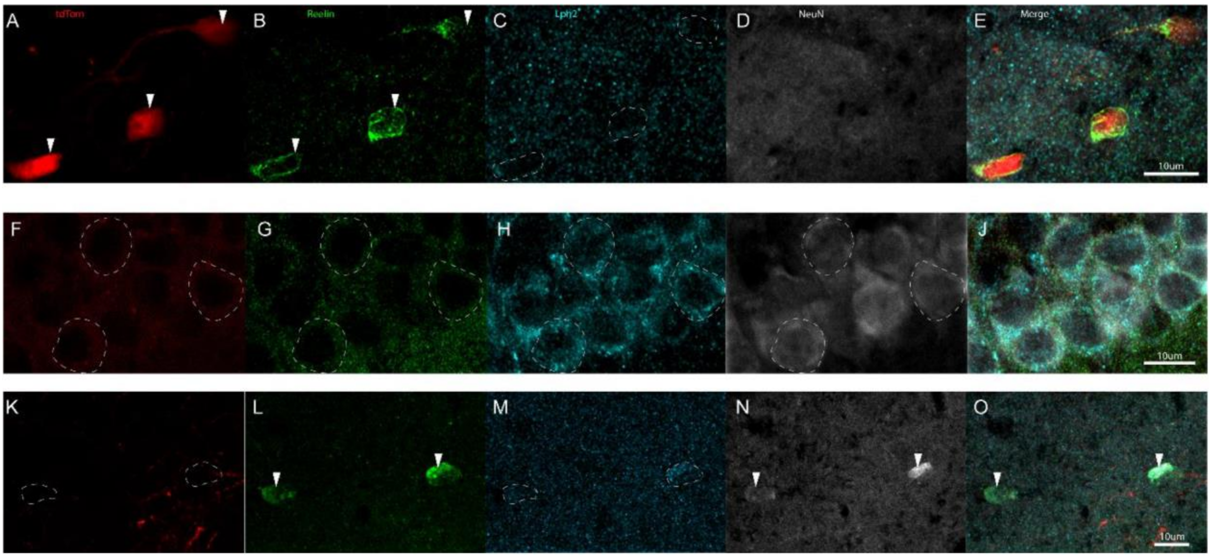


Confocal images of colocalization of TdTomato, Lphn2 and reelin. First row depicting CR cells expression of TdTomato (A), reelin (B), Lphn2 (C), NeuN (D), and merged (E). Second row depicting pyramidal cell expression of TdTomato (F), reelin (G), Lphn2 (CH, NeuN (I), and merged (J). Third row depicting interneurons expression of TdTomato (K), reelin (L), Lphn2 (M), NeuN (N), and merged (O).

**3. For Figure 2C – was there correction for multiple comparisons applied? If not why?**
We thank the reviewer for bringing this issue to our attention. Our original choice was to follow current literature and perform a Mann-Whitney test on each ring with no correction (Bose et al., 2010; Florio et al., 2012; Hamad et al., 2014; Hamad et al., 2021; Jacobs et al., 2016; Riva et al., 2019). Even though the distribution of cells is the same and tested several times, the subsample (dendritic segments) that are tested for each ring is only tested once. The Mann-Whitney-U test itself is not influenced by the tests that were done before.
However, in order to avoid the issue of multiple comparisons altogether, we decide to modify our approach. We have now removed the testing on each ring and only analyzed the area under the curve (showed now in Panel 2D). This allows for a single point of comparison using the Mann-Whitney-U test. We believe that this new approach does not lead to a loss of information, as the curves clearly show that the changes in dendritic complexity are not limited to a specific layer (see updated lines: 140-143 & 626-647).

**4. For Figure 4 – It would seem more logical to present the p values for the ANCOVA tests on each graph as well as or even instead of the p values for the regression model of each condition in isolation. At the moment it's hard to quickly compare the statistical differences between conditions from the figure.**

The reviewer makes a valid point as there are a lot of statistics in this figure. We focused on the regression model as that is the main basis of our interpretation, but we added the p values of the intercept and slope from the ANCOVA to the figure for clarity.

******* Reviewer 2 Advance Summary and Potential Significance to Field: The authors make use of an elegant experimental paradigm to get rid of a crucial developmental cell population, CR cells, and investigate their role in circuit formation. There is a clear experimental pipeline that details the sequence of findings that bring to the final proposal. The authors are knowledgeable in this matter and their results/interpretations are mostly well supported (except for the cases stated below). I truly acknowledge the value of the discussion section, which truly compares this novel data to many recent and classic articles in the field. Altogether, I am convinced this article would bring many novel concepts and results to the field, and would be greatly appreciated and referenced, not only by researchers working directly on hippocampus or Cajal-Retzius, but also in general brain and cortical development as well as circuit formation. Cajal-Retzius cells are making much more than originally expected. Reviewer 2 Comments for the**

© 2024. Published by The Company of Biologists under the terms of the Creative Commons Attribution License (https://creativecommons.org/licenses/by/4.0/).

**Author: In conclusion, this is a good piece of research that conducts experimentation in a logic fashion. The interpretation of their results is adequate, and their conclusion are mostly well supported by their data. There are only some comments and suggestions that I would like the authors to address prior publication.**

**•My main concern is the direct relation of the loss of CR cells to the malformed hippocampal circuitry. The authors unambiguously identify that in absence of CR cells fewer synapses are made on CA1 neurons at the SLM level. What is not clear to me is the neuronal origin of these lacking synapsis in the SLM. Surely CR cells may contact CA1 cells at this layer, and it is obvious that when CR cells are not present, their synapses are gone. If the origin of the missing synapsis is primarily CR cells, their loss would not be affecting hippocampal circuitry, but only a dull, direct connection CR-CA1. The authors discuss that the origin of these synapsis are entorhinal neurons, but there is no evidence for this, neither coming from the literature nor from direct experimentation. In my opinion, the entorhinal origin of SLM synapsis must be proven in a more convincing way, by either direct tracing or comprehensive literature on the synapsis coming from CR cells. Until then, the lack of CR cells may seem to be affecting only their own missing connections.**

The reviewer is correct in that CR cells contact pyramidal neurons directly. However, we would argue that the effect we see is not due to this connection as at early timepoints (P22) we see an increased spine density before a decrease in density at later timepoints (P35) in the SLM (please see figure 4A). We know that CR cells have been successfully ablated by P22, as figure 1G demonstrates a drastic reduction of CR cells at P15. We argue that the effective loss of spines at the later time points analyzed is therefore likely not due to this direct connection. Moreover, we know from previous work that the direct connection between CR cells and pyramidal cells is rather rare, as optogenetic stimulation of CR cells elicits a response only in ~14% of the recorded pyramidal cells (Quattrocolo & Maccaferri, 2014). Similar experiments studying the EC to CA1 pyramidal cells connections with optogenetic simulation have demonstrated an incredibly high level of connectivity (>90%) (Li et al., 2017). In addition, electron microscopy showed that 85% of the connections made by CR cells are on dendritic shafts, and only the remaining 15% are on spines (Anstötz et al. 2015), arguing for a strong bias of CR cells to target interneurons. Based on both the electrophysiological experiments, and those of electron microscopy we can be fairly confident that the CR-cells to pyramidal cells connectivity is probably too small to make the impact we are seeing with our morphological analysis. We have elaborated on these points in the discussion (Lines: 178-180 & 526-545).

A direct projection from the entorhinal cortex to the hippocampus, and specifically from layer 3 to CA1 pyramidal cells is well established in the literature (Canto et al., 2008; Dudman et al., 2007; Ito & Schuman, 2012; Li et al., 2017; Masurkar et al., 2017; Naber et al., 2001; Suh et al., 2011; Witter, 1993). A paper from 2017 from the Sudhof lab (Anderson et al., 2017) showed that Lhpn2 conditional KO specifically affects the projection from the EC to CA1, with selective loss of spine in SLM. Our basis for suggesting a disruption of the entorhinal-hippocampal circuit derives from this study, as well as subsequent work from both the Sudhof as well as the Luo lab (Pederick et al., 2021; Sando & Südhof, 2021). If we exclude CR cells and EC fibers, the only other major glutamatergic input in SLM derives from the thalamus, but those fibers do not contact CA1 pyramidal cells (Andrianova et al., 2021; Leprince et al., 2023). While a disruption of the circuit could be interpreted as the EC axons missing its SLM target, we find this possibility unlikely, as there are still some spines present in the SLM and we see no increase in spine in any of the other hippocampal layers (figure 4BC). Indeed, we see a decrease in density of spines also in SR (-45% thin spines), a layer targeted by completely different projections (mainly axons from CA3 pyramidal cells).

While we see the point of the reviewer in proposing the tracing experiment, we chose to focus on other elements, such as Lphn2, in this paper because we are not convinced the tracing experiments could lead us to consistent results. There are several caveats in comparing two groups using an anterograde tracer. First, there is always variability in the size and specific location of the injection site. Moreover, comparison of anterograde tracing across animals is affected by the topography of the projection, which results in an increased number of animals needed. Second, we would need to perform injections at a relatively young age (P15), therefore the specificity of the injection site will likely be reduced, aggravating the issue of topography of projections. In relation

© 2024. Published by The Company of Biologists under the terms of the Creative Commons Attribution License (https://creativecommons.org/licenses/by/4.0/).

to this issue, we need to consider the topography of the connections between EC and CA1, with LEC fibers reaching more distal regions and MEC targeting proximal CA1. Any variability in the injection site or level of the infection will make a quantification and comparison between the two conditions very difficult and likely lead to potentially confounding results, not justifying, in our opinion, the high number of animals that would be needed to perform this experiment.

**•Are there any hippocampal populations not directly connected to CR cells? If so, I would suggest to quickly check whether their synaptogenesis is somehow altered as well.**
From previous research we know that CR cells primarily have arborizations within the SLM and the molecular layer of the dentate gyrus (Anstötz et al., 2016) Therefore we can be fairly confident that the effects we see from our morphological analysis in the stratum radiatum and stratum oriens and the changes in spine density in radiatum (a reduction of 45%) are not due to a loss of direct contact between the pyramidal cell and CR cells as the CR cell axon does not project there. As the other major population residing in SLM are the neurogliaform GABAergic interneurons, which are highly interconnected to CR cells, it would be difficult to assess changes in synaptogenesis in other cell types in SLM, as, in this case, the loss of synaptic contact could be due directly to the missing CR cells. We elaborated more on the topic of connectivity in this version of the discussion (Lines: 526-545).

**•Some relevant experiments are missing the comparison to the total control, which is the uninfected hippocampus. Because maybe the simple viral infection without CR loss may affect somehow the circuitry or even the survival of some CR cells. Is there any difference between total control and infected animals in the number of CR cells and their marker expression (Fig. 1)? And in layer-specific dendritic spine density of CA1 pyramidal cells (Fig. 3 and 4)?**

We apologize if this point has not been sufficiently clarified in the text. We did quantify the density of CR cells in an uninjected control (Pde1c-Cre;tdTomato at P30) (see lines 104-106 and figure 1G *right*). These results show no significant differences between the injected control and the uninjected control in terms of CR cell density. Therefore, the effects we see are likely not due to the injection per se, but we will take measures to further clarify this in the text (Line 105). We did not perform a morphological analysis on the uninjected controls, but we know that the spine density in our control group is comparable to what has been shown in other studies of age-matched controls (Anderson et al., 2017), supporting the idea that the injection per se does not cause significant alterations in the morphology of pyramidal cells. We included this in the results (Lines 190-191).

**•The selection of markers of CR cells tested in fig. 5C is based in an article (Jimenez and Moreno, 2021) which is likely a bad choice. This article studies non-mammalian animals, on which the existence of CR cells is still under debate. There are plenty of great articles based on mammalian CR cells from which to extract a list of actual markers. Those on the list in Fig. 5C may be (or not) the same.**

We realized this was not the best choice to pick CR cells markers, as it is known that species-specific genes are variable across conserved cell types. We have now incorporated markers from the Pangalao database of Cajal-Retzius cell markers, the Allen Brain Atlas Transcriptomic dataset, and other CR cell markers as supported by literature in Mus musculus and Homo sapiens and rectify potential mistypes or incorrect homologs in the marker list (see revised figure 5C).

**•In fig. 8C there is a clear change in the expression of Lphn2 at the hilus level and its surroundings. Could the authors mention this in the article and speculate on its origin and consequences?**
We are happy to speculate on this, however, these results in the hilus were not significant and when taking a closer look at the densitometric analysis in Fig 8D, it is quite clear that Lphn2 changes in the DG are not as consistent between genotypes as in the SLM. Therefore, we have not discussed these changes so as not to present misleading statements alongside our observations of changes in the CA1, as this latter region is the main focus of the paper. We provide a more in-depth assessment of Lphn2 with respect to our new staining (see updated figure 8E-F).

**•CR cells are born to die early in postnatal development, in great numbers in the cortex. It is not clear at what postnatal age post-infection CR cells die due to this experimental paradigm. It**

© 2024. Published by The Company of Biologists under the terms of the Creative Commons Attribution License (https://creativecommons.org/licenses/by/4.0/). 

**is relevant because they may die early and affect circuit formation from very early on: changes could be there, visible, from very early after infection. Or they may take some days to die, and so the aberrant formation of the circuit could be a late event. I suggest the authors to briefly assess CR cell loss 3 and 7 days after infection, to help draw these conclusions.**

While we agree that knowledge of the exact timepoint the CR cells die after infection would be interesting to assess, we believe that this experiment would not bring major insight to the current story. At the moment, all the alterations we have identified are linked to spines and synapses. Evaluation of death at an earlier timepoint would need to be matched by the quantification of some parameters (not related to spines, as they are not present really until the second postnatal week) to determine when the consequences of CR cells ablation become detectable. At the moment, we have not yet identified such a parameter.

****** Reviewer 3 Advance Summary and Potential Significance to Field: In the present study Lynneberg Glaerum and collegues induced premature death of hippocampal Cajal- Retzius cells in the early postnatal mouse hippocampus by viral vector injection. They characterized morphological changes in dendrite complexity as well as spine density of neurons of CA1 pyramidal neurons in these mice. Moreover, they characterized changes in gene regulatory networks, more specifically, they found that expression of Latrophilin-2 was significantly changed. The study has the potential to provide an important advance to the field if morphological circuit alterations of specific fiber projections could be shown by the authors.**
**Reviewer 3 Comments for the Author: While a critical role for Cajal-Retzius cells in the establishment of the hippocampal circuit is proposed in the title of the study, unfortunately no morphological evidence for changed or developmentally challenged fiber projections is shown. Presenting reduced dendritic complexity is not sufficient for this claim, since this is also observed in (naturally or induced) reelin deficient mice, which nonetheless have largely normal circuits in hippocampus and neocortex. Thus, showing an altered hippocampal circuit or an effect on the entorhino-hippocampal projection, would largely improve the study. Of note, the authors cite the Del Rio et al. (1997) who showed reduced ingrowth of entorhinal fibers in the hippocampus after pharmacological ablation of Cajal-Retzius cells in a co-culture system (see Discussion, line 496- 97 of present manuscript). While the latter study has demonstrated this in vitro, the present in vivo model has the great potential to explore if these changes actually also happen in vivo after ablation of Cajal-Retzius cells.**
**Thus, if effects on the development of fiber projections could be shown in vivo, for example by using tracing techniques, the implications would be far- reaching and therefore largely improve the study.**

We greatly appreciate the reviewer's insightful suggestions, as performing tracing experiments from the entorhinal cortex is something we have discussed amongst ourselves. Though, we chose to focus on other elements, such as Lphn2, in this paper because we are not convinced the tracing experiments could lead us to consistent results. There are several caveats in comparing two groups using an anterior tracer. First, there is always variability in the infection. Second, we would need to perform injections at a relatively young age (P15), therefore the specificity of the injection site will likely be reduced. In relation to this issue, we need to consider the topography of the connections between EC and CA1, with LEC fibers reaching more distal regions and MEC targeting proximal CA1. Any variability in the injection site or level of the infection will make a quantification and a comparison between the two conditions very difficult and likely lead to a potentially confounding negative result, not justifying, in our opinion, the high number of animals that would be needed to perform this experiment.
Additionally, the role CR cells have in axonal pathfinding is mostly attributed to their prenatal functions (Del Río et al., 1997) as the EC axons mostly innervate the hippocampus before birth (~E16; Super et al. 1998). The Del Rio et al paper from 1997 performs a co-culture experiment to study the *re*-innervation of the hippocampus by EC fibers and observe that EC fibers are not able to invade the SLM in the absence of CR cells, but no mistargeting was present.
With all those things in mind, we focused on Lphn2, which is a protein associated with the establishment of EC-hippocampal circuit and its maintenance (Anderson et al. 2017). As we see a difference in Lphn2 (both mRNA and staining for the protein) we argue that this circuit is affected by CR cell ablation, but we make no claim that it means the EC fibers are diverted. Likely they are not, as we don't see an increase in spine density in the other layers of the hippocampus (stratum

© 2024. Published by The Company of Biologists under the terms of the Creative Commons Attribution License (https://creativecommons.org/licenses/by/4.0/).

radiatum and stratum oriens). A likely possibility is that the maintenance of the EC to CA1 connections are impaired, with a potential late reduction in arborization. This could explain the initial increase in Lphn2 at P15 before the reduction at P30. We made sure to speculate on circuit disruptions upon CR cells ablation, for better clarity (lines: 526-530). In addition, we would like to point out the alterations in spine density observed in radiatum, where completely different cell types and projections reside (mainly inputs from CA3 pyramidal cells), and where there are no CR cells nor their axons, suggesting that different hippocampal microcircuits are affected by CR cells ablation (see lines 178-180 & 531-545)

**minor: the citation Ramon y Cajal et al is not complete in the reference list, for instance the publisher is missing.**

We apologize for the mistake, and we have corrected it.

**References**

Anderson, G. R., Maxeiner, S., Sando, R., Tsetsenis, T., Malenka, R. C., & Südhof, T. C. (2017). Postsynaptic adhesion GPCR latrophilin-2 mediates target recognition in entorhinal-hippocampal synapse assembly. *J Cell Biol*, *216*(11), 3831-3846. https://doi.org/10.1083/jcb.201703042

Andrianova, L., Brady, E. S., Margetts-Smith, G., Kohli, S., McBain, C. J., & Craig, M. T. (2021). Hippocampal CA1 pyramidal cells do not receive monosynaptic input from thalamic nucleus reuniens. *BioRxiv*, 2021.2009. 2030.462517.

Bose, M., Muñoz-Llancao, P., Roychowdhury, S., Nichols, J. A., Jakkamsetti, V., Porter, B., Byrapureddy, R., Salgado, H., Kilgard, M. P., Aboitiz, F., Dagnino-Subiabre, A., & Atzori, M. (2010). Effect of the environment on the dendritic morphology of the rat auditory cortex. *Synapse*, *64*(2), 97- 110. https://doi.org/10.1002/syn.20710

Canto, C. B., Wouterlood, F. G., & Witter, M. P. (2008). What does the anatomical organization of the entorhinal cortex tell us? *Neural Plast*, *2008*, 381243. https://doi.org/10.1155/2008/381243

Dudman, J. T., Tsay, D., & Siegelbaum, S. A. (2007). A role for synaptic inputs at distal dendrites: instructive signals for hippocampal long-term plasticity. *Neuron*, *56*(5), 866-879. https://doi.org/10.1016/j.neuron.2007.10.020

Florio, M., Leto, K., Muzio, L., Tinterri, A., Badaloni, A., Croci, L., Zordan, P., Barili, V., Albieri, I., Guillemot, F., Rossi, F., & Consalez, G. G. (2012). Neurogenin 2 regulates progenitor cell-cycle progression and Purkinje cell dendritogenesis in cerebellar development. *Development*, *139*(13), 2308-2320. https://doi.org/10.1242/dev.075861

Hamad, M. I., Jack, A., Klatt, O., Lorkowski, M., Strasdeit, T., Kott, S., Sager, C., Hollmann, M., & Wahle, P. (2014). Type I TARPs promote dendritic growth of early postnatal neocortical pyramidal cells in organotypic cultures. *Development*, *141*(8), 1737-1748. https://doi.org/10.1242/dev.099697

Hamad, M. I. K., Petrova, P., Daoud, S., Rabaya, O., Jbara, A., Melliti, N., Leifeld, J., Jakovčevski, I., Reiss, G., Herz, J., & Förster, E. (2021). Reelin restricts dendritic growth of interneurons in the neocortex. *Development*, *148*(17). https://doi.org/10.1242/dev.199718

Ito, H. T., & Schuman, E. M. (2012). Functional division of hippocampal area CA1 via modulatory gating of entorhinal cortical inputs. *Hippocampus*, *22*(2), 372-387. https://doi.org/10.1002/hipo.20909

Jacobs, S., Cheng, C., & Doering, L. C. (2016). Hippocampal neuronal subtypes develop abnormal dendritic arbors in the presence of Fragile X astrocytes. *Neuroscience*, *324*, 202-217. https://doi.org/10.1016/j.neuroscience.2016.03.011

Leprince, E., Dard, R. F., Mortet, S., Filippi, C., Giorgi-Kurz, M., Bourboulou, R., Lenck-Santini, P.-P., Picardo, M. A., Bocchio, M., & Baude, A. (2023). Extrinsic control of the early postnatal CA1 hippocampal circuits. *Neuron*, *111*(6), 888-902. e888.

Li, Y., Xu, J., Liu, Y., Zhu, J., Liu, N., Zeng, W., Huang, N., Rasch, M. J., Jiang, H., Gu, X., Li, X., Luo, M., Li, C., Teng, J., Chen, J., Zeng, S., Lin, L., & Zhang, X. (2017). A distinct entorhinal cortex to hippocampal CA1 direct circuit for olfactory associative learning. *Nature Neuroscience*, *20*(4), 559-570. https://doi.org/10.1038/nn.4517

© 2024. Published by The Company of Biologists under the terms of the Creative Commons Attribution License (https://creativecommons.org/licenses/by/4.0/).

Masurkar, A. V., Srinivas, K. V., Brann, D. H., Warren, R., Lowes, D. C., & Siegelbaum, S. A. (2017). Medial and Lateral Entorhinal Cortex Differentially Excite Deep versus Superficial CA1 Pyramidal Neurons. *Cell Rep*, *18*(1), 148-160. https://doi.org/10.1016/j.celrep.2016.12.012

Naber, P. A., Lopes da Silva, F. H., & Witter, M. P. (2001). Reciprocal connections between the entorhinal cortex and hippocampal fields CA1 and the subiculum are in register with the projections from CA1 to the subiculum. *Hippocampus*, *11*(2), 99-104. https://doi.org/https://doi.org/10.1002/hipo.1028

Pederick, D. T., Lui, J. H., Gingrich, E. C., Xu, C., Wagner, M. J., Liu, Y., He, Z., Quake, S. R., & Luo, L. (2021). Reciprocal repulsions instruct the precise assembly of parallel hippocampal networks. *Science*, *372*(6546), 1068-1073. https://doi.org/10.1126/science.abg1774

Riva, M., Genescu, I., Habermacher, C., Orduz, D., Ledonne, F., Rijli, F. M., Lopez-Bendito, G., Coppola, E., Garel, S., Angulo, M. C., & Pierani, A. (2019). Activity-dependent death of transient Cajal- Retzius neurons is required for functional cortical wiring. *Elife*, *8*. https://doi.org/10.7554/eLife.50503

Sando, R., & Südhof, T. C. (2021). Latrophilin GPCR signaling mediates synapse formation. *Elife*, *10*. https://doi.org/10.7554/eLife.65717

Suh, J., Rivest, A. J., Nakashiba, T., Tominaga, T., & Tonegawa, S. (2011). Entorhinal Cortex Layer III Input to the Hippocampus Is Crucial for Temporal Association Memory. *Science*, *334*(6061), 1415-1420. https://doi.org/10.1126/science.1210125

Witter, M. P. (1993). Organization of the entorhinal-hippocampal system: a review of current anatomical data. *HIPPOCAMPUS-NEW YORK-CHURCHILL LIVINGSTONE-*, *3*, 33-33.

---

Second decision letter

MS ID#: DEVELOP/2023/202236

MS TITLE: Postnatal persistence of hippocampal Cajal-Retzius cells has a critical role in the establishment of the hippocampal circuit

AUTHORS: Ingvild Lynneberg Glærum, Keagan Dunville, Kristian Moan, Maike Krause, Nicola Pietro Montaldo, Hinako Kirikae, Maximiliano Jose Nigro, Pål Sætrom, Barbara van Loon, and Giulia Quattrocolo
ARTICLE TYPE: Research Article

I am delighted to tell you that your manuscript has been accepted for publication in Development, pending our standard ethics checks.

Reviewer 1

*Advance summary and potential significance to field*

The authors have done an excellent and comprehensive job of addressing all of the questions and suggestions posed. It is a significant and elegant study that will add greatly to the field.

*Comments for the author*

I have no further comments or suggestions, all of my original questions and comments have been adequately addressed.

Reviewer 2

*Advance summary and potential significance to field*

The authors make use of an elegant experimental paradigm to get rid of a crucial developmental cell population, CR cells, and investigate their role in circuit formation. There is a clear

© 2024. Published by The Company of Biologists under the terms of the Creative Commons Attribution License (https://creativecommons.org/licenses/by/4.0/).

experimental pipeline that details the sequence of findings that bring to the final proposal. The authors are knowledgeable in this matter and their results/interpretations are mostly well supported (except for the cases stated below). I truly acknowledge the value of the discussion section, which truly compares this novel data to many recent and classic articles in the field. Altogether, I am convinced this article would bring many novel concepts and results to the field, and would be greatly appreciated and referenced, not only by researchers working directly on hippocampus or Cajal-Retzius, but also in general brain and cortical development as well as circuit formation. Cajal- Retzius cells are making much more than originally expected.

In conclusion, this is a good piece of research that conducts experimentation in a logic fashion. The interpretation of their results is adequate, and their conclusion are well supported by their data.

*Comments for the author*

The authors clarified all my concerns and improved the article for a better reading. I have no further concerns.

Reviewer 3

*Advance summary and potential significance to field*

in my opinion, the study has the potential to give a significant impulse to the field

*Comments for the author*

the authors sufficiently addressed all criticisms either with new experiments or with good arguments explaining why they did not exactly follow the reviewer(s) line - I have no further criticisms.

© 2024. Published by The Company of Biologists under the terms of the Creative Commons Attribution License (https://creativecommons.org/licenses/by/4.0/).
